# Supplementary material for: A Nuclear Long Non-Coding RNA LINC00618 Accelerates Ferroptosis in a Manner Dependent upon Apoptosis
Source: Mol Ther. 2020 Sep 20;29(1):263–74. doi: 10.1016/j.ymthe.2020.09.024 (PMC7791008; doi:10.1016/j.ymthe.2020.09.024)
Supplement: Document S1. Figures S1–S6 and Tables S1–S3 [file mmc1.pdf]

YMTHE, Volume *29*

Supplemental Information

A Nuclear Long Non-Coding RNA LINC00618 Accelerates Ferroptosis in a Manner Dependent upon Apoptosis

Zuli Wang, Xiaowen Chen, Na Liu, Ying Shi, Yating Liu, Lianlian Ouyang, Samantha Tam, Desheng Xiao, Shuang Liu, Feiqiu Wen, and Yongguang Tao

Supplementary Figures

Figure S1

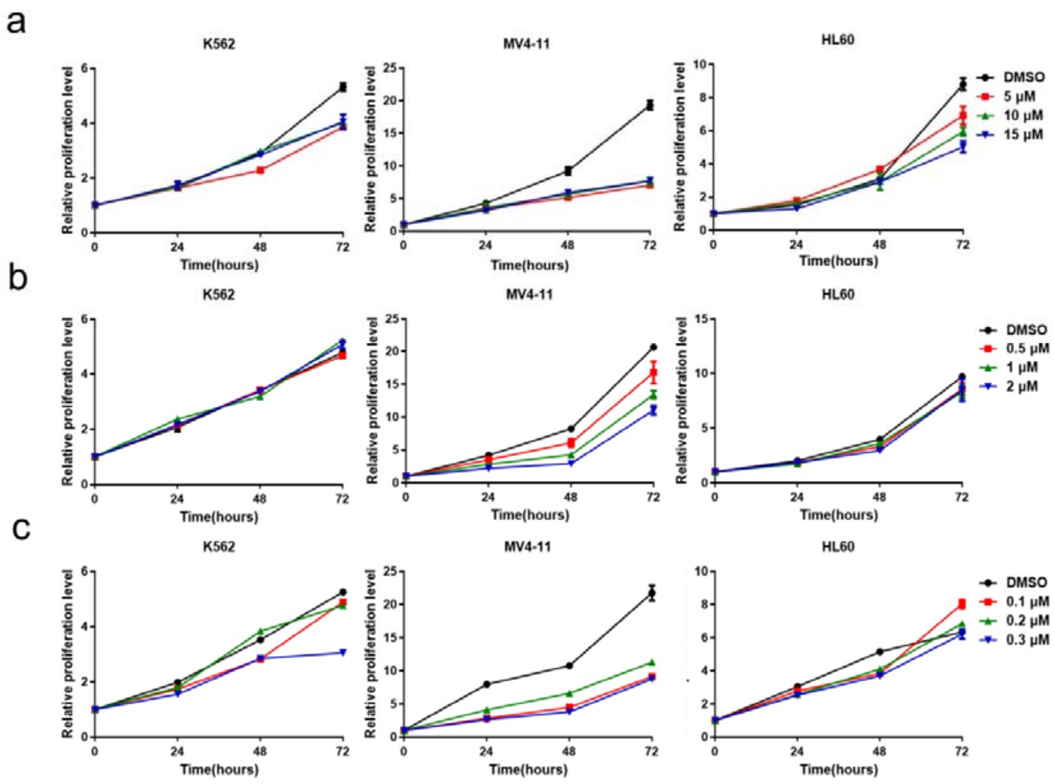

Figure. S1. 6-mercatopurine monohydrate, Imatinib Mesylatem and AZD9496 affecting cell growth.

a The relative proliferation levels of K562, HL60 and MV4-11 cells checked by MTS assays with the indicated doses of 6-Mercatopurine Monohydrate treatment. DMSO served as control. **b** The relative proliferation levels of K562, HL60 and MV4-11 cells checked by MTS assays with the indicated doses of Imatinib Mesylate treatment. DMSO served as control. **c** The relative proliferation levels of K562, HL60 and MV4-11 cells checked by MTS assays with the indicated doses of AZD9496 treatment. DMSO served as control.

Figure S2

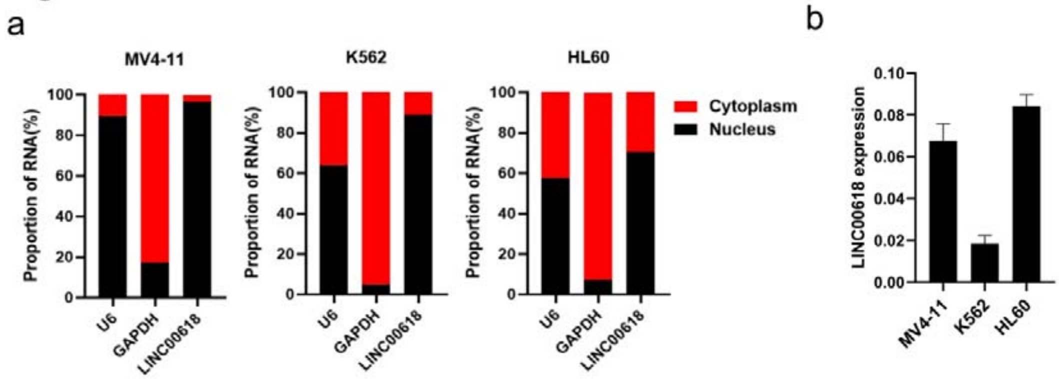

Figure. S2. The expression and localization of LINC00618 in MV4-11, K562 and HL60 cells.

a The RNA expression level of LINC00618 in MV4-11, K562 and HL60 cells. **b** The LINC00618 expression level in the nuclear and cytosolic fractions derived from K562 cells and HL60 cells

Figure S3

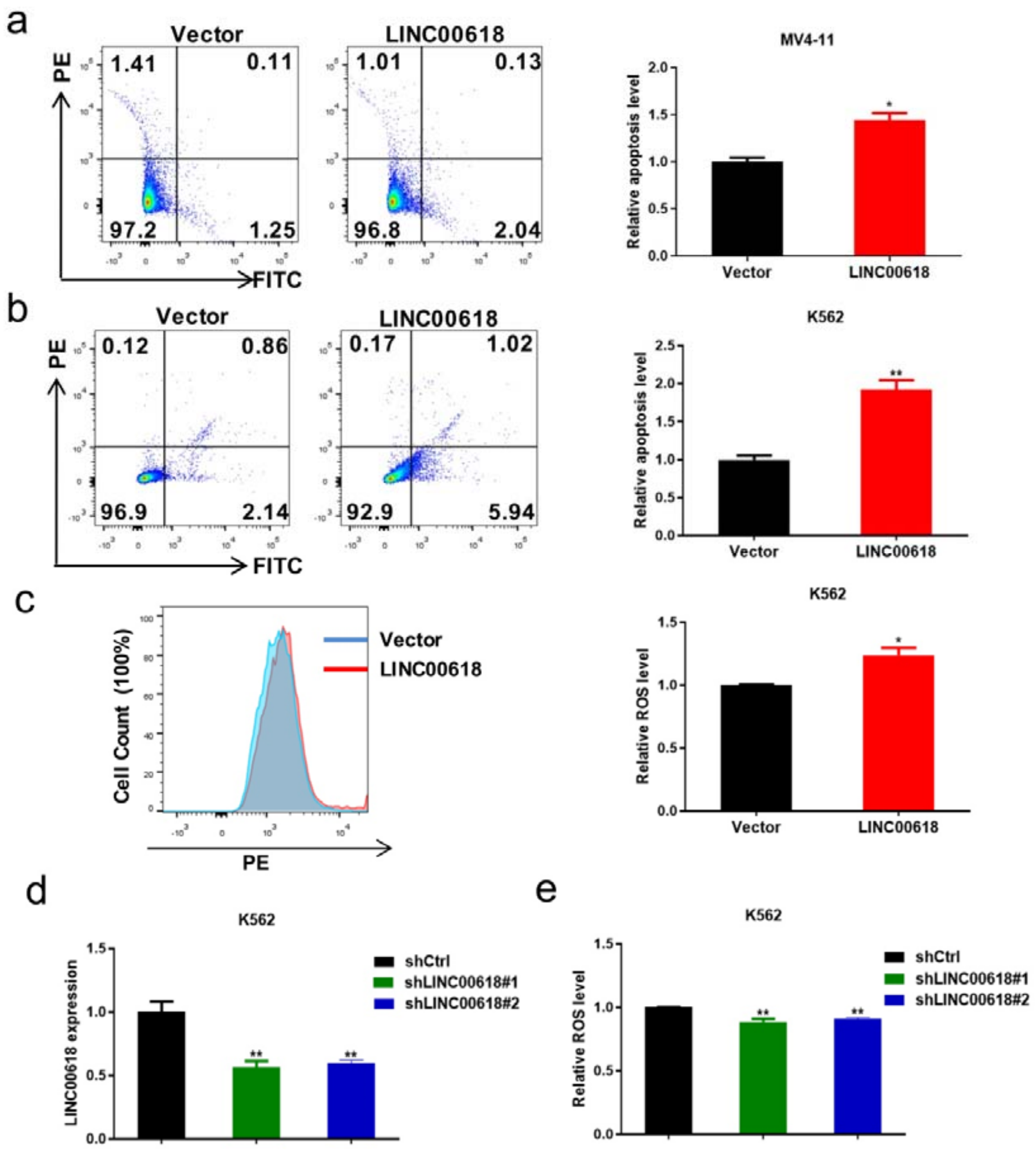

Figure. S3. The role of LINC00618 in promoting cell apoptosis and ferroptosis.

a Flow cytometry and statistical analysis showed that LINC00618 promoted apoptosis in MV4-11 cells stably overexpressing LINC00618. **b** Flow cytometry and statistical analysis showed that LINC00618 promoted apoptosis in K562 cells stably overexpressing LINC00618. **c** Flow cytometry and statistical analysis of ROS level in K562 cells stably overexpressing LINC00618. **d** qRT-PCR analyses of stably knockdown vector and LINC00618 in K562 cells. **e** Flow cytometry and statistical

analysis of ROS level in LINC00618-knockdown K562 cells.

Data are shown as the mean ± SEM; $n \geq 3$ independent experiments, two-tailed Student's t-test: ns nonsignificant ($p > 0.05$), *$P < 0.05$, **$P < 0.01$, ***$P < 0.001$.

Figure S4

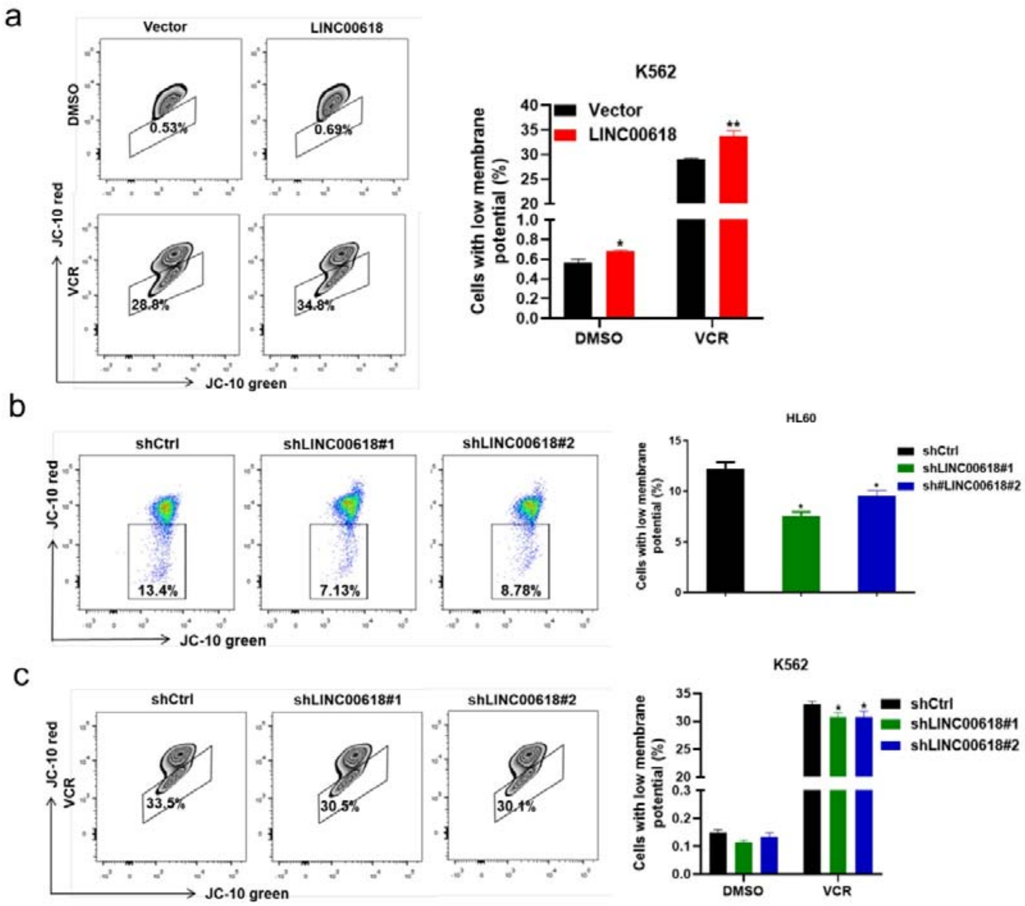

Figure. S4. The role of LINC00618 in mitochondrial membrane potential.

a Flow cytometry and statistical analysis of mitochondrial membrane potential using JC-10 in LINC00618-overexpressed K562 cells treated with VCR. DMSO served as control. **b** Flow cytometry and statistical analysis of mitochondrial membrane potential using JC-10 in LINC00618-konckdown HL60 cells. **c** Flow cytometry and statistical analysis of mitochondrial membrane potential using JC-10 in LINC00618-knockdown K562 cells treated with VCR. DMSO served as control.

Figure S5

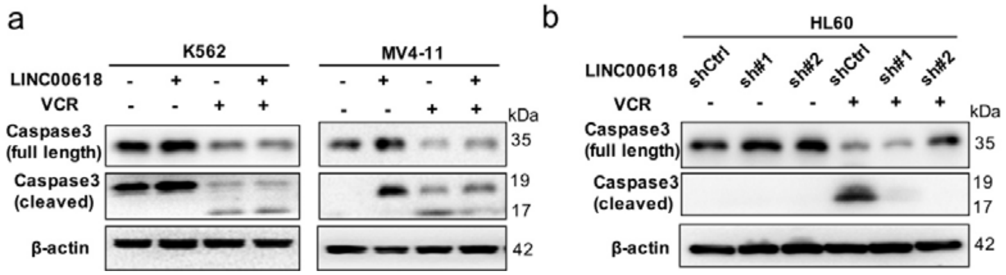

Figure. S5. The effect of LINC00618 on apoptosis related proteins.

a Expression level of apoptosis related proteins were measured by western blot in K562 and MV4-11 cells stably overexpressing LINC00618. **b** Expression level of apoptosis related proteins were measured by western blot in HL60 cells stably interfering LINC00618.

Figure S6

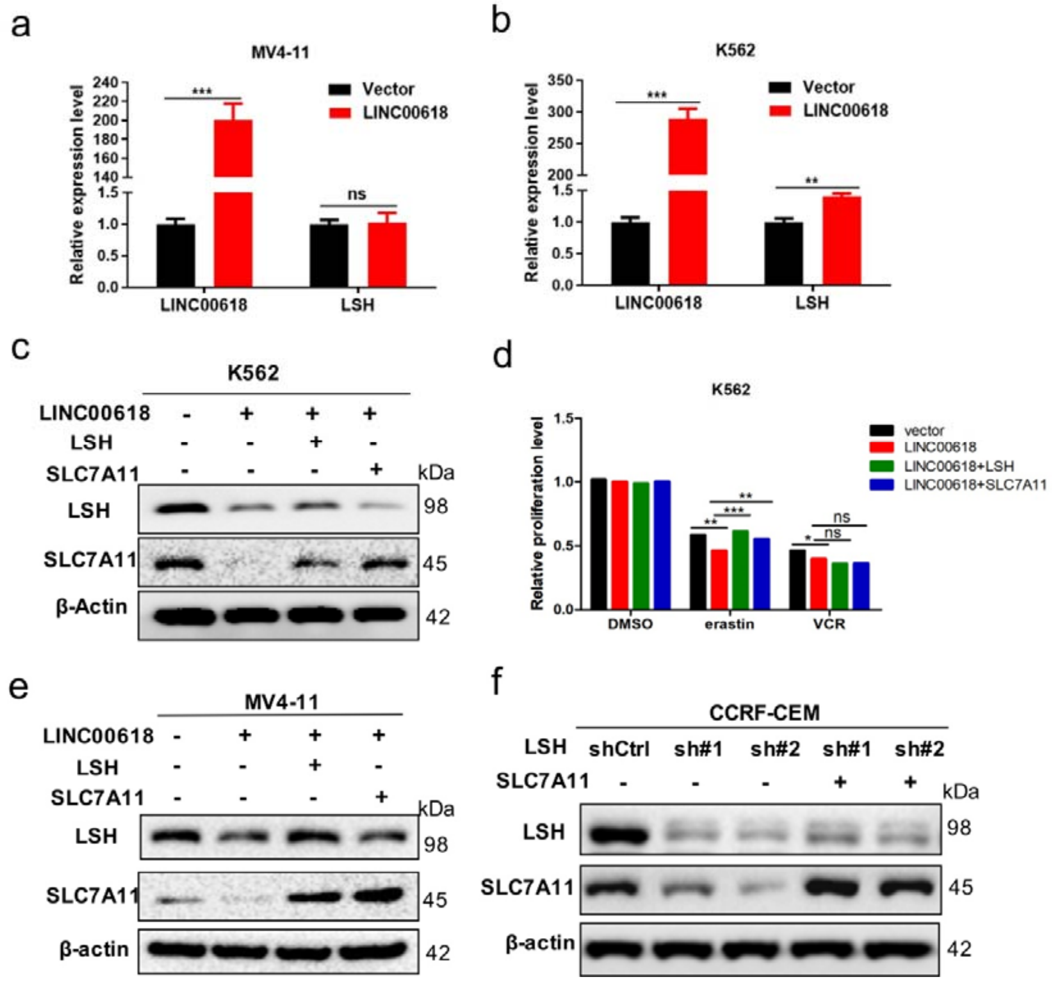

Figure. S6. SLC7A11 is regulated by LINC00618 through LSH.

a qRT-PCR analyses of LSH in MV4-11 cells stably overexpressing LINC00618. **b** qRT-PCR analyses of LSH in K562 cells stably overexpressing LINC00618. **c** Western blot analyses of LSH and SLC7A11 in K562 cells stably overexpressing LINC00618 with LSH or SLC7A11 overexpression. **d** MTS assay analyses of relative proliferation levels of K562 cell lines with erastin or VCR treatment from **c.** DMSO served as control. **d W**estern blot analyses of LSH and SLC7A11 in MV4-11 cells stably overexpressing LINC00618 with LSH or SLC7A11 overexpression. **e** Western blot analyses of LSH and SLC7A11 in CCRF-CEM cells after the knockdown of LSH

with SLC7A11 overexpression.

Supplementary Tables

Table S1. Primer sequences used for RT-PCR

Name	Full Name	Gene ID	sequence	Product Size(bp)
LINC00618	long intergenic non-protein coding RNA 618	145249	F: AAGGGGTGGGGACACGTTG<br>R: TCTCTCTCCGTCAGCCTCAG	140
LSH	Lymphoid-specific helicase	3070	F: GATTTTGGATCGAATGCTGCCAG<br>R: ATGGACCCATCAAGCCTGCTGA	141
SLC7A11	solute carrier family 7 member 11	23657	F: TCCTGCTTTGGCTCCATGAACG<br>R: AGAGGAGTGTGCTTGCGGACAT	122
GPX4	glutathione peroxidase 4	2879	F: ACAAGAACGGCTGCGTGGTGAA<br>R: GCCACACACTTGTGGAGCTAGA	126
ACSL4	acyl-CoA synthetase long chain family member 4	2182	F: GCTATCTCCTCAGACACACCGA<br>R: AGGTGCTCCAACTCTGCCAGTA	139
P53	Tumor protein p53	7157	F: CCTCAGCATCTTATCCGAGTGG<br>R: TGGATGGTGGTACAGTCAGAGC	128
CS	citrate synthase	1431	F: CACAGGGTATCAGCCGAACCAA<br>R: CCAATACCGCTGCCTTCTCTGT	127
ATP5G3	ATP synthase membrane subunit c locus 3	518	F: GCAACAGTAGGAGTGGCTGGTT<br>R: GCTTCAGACAAGGCAAATCCCAG	137
CBS	cystathionine-beta-synthase	875	F: CATTGCCAGGAAGCTGAAGGAG<br>R: TTCCACCTCGTAGGTTGTCTGC	124
HMGCR	3-hydroxy-3-methylglutaryl -CoA reductase	3156	F: GACGTGAACCTATGCTGGTCAG<br>R: GGTATCTGTTTCAGCCACTAAGG	119
LPCAT3	lysophosphatidylcholine acyltransferase 3	10162	F: CAGGATACCTGGTCTGCTTCCA<br>R: TGAAGAGCCAGTGGATGGTCTG	162
TFRC	transferrin receptor	7037	F: ATCGGTTGGTGCCACTGAATGG<br>R: ACAACAGTGGGCTGGCAGAAAC	131

Table S2. Primer sequences used for ChIP

Name	Sequence	Product size(bp)
SLC7A11#1	F: AGTTGGTGTGACAGGCAGGCGCTTA	122
	R: TCTCAATTCTCCACCTCCTCGTTCC	
SLC7A11#2	F: GGAACGAGGAGGTGGAGAATTGAGA	123
	R: CTCAGCGCTATAGTGTTCACAGGTG	

Table S3. Probe sequences used for ChIRP

Name	Sequence	modification
LINC00618#1	CACCATGGATGCAACGTGTC	3`Biotin-TEG
LINC00618#2	TTCATCTCTGGAAACTGCCC	3`Biotin-TEG
LINC00618#3	TGGCTTCGATCCAGAGTGTC	3`Biotin-TEG
