# Supplementary material for: A Nuclear Long Non-Coding RNA LINC00618 Accelerates Ferroptosis in a Manner Dependent upon Apoptosis
Source: Mol Ther. 2020 Sep 20;29(1):263–74. doi: 10.1016/j.ymthe.2020.09.024 (PMC7791008; doi:10.1016/j.ymthe.2020.09.024)
Supplement: Document S2. Article plus Supplemental Information [file mmc2.pdf]

Molecular Therapy
Original Article

A Nuclear Long Non-Coding RNA LINC00618 Accelerates Ferroptosis in a Manner Dependent upon Apoptosis

Zuli Wang,[1,2,9] Xiaowen Chen,[3,4,9] Na Liu,[1,2] Ying Shi,[1,2] Yating Liu,[1,2] Lianlian Ouyang,[1,2] Samantha Tam,[5] Desheng Xiao,[1,6] Shuang Liu,[7] Feiqiu Wen,[3,4] and Yongguang Tao[1,2,8]

[1]Key Laboratory of Carcinogenesis and Cancer Invasion, Ministry of Education, Department of Pathology, Xiangya Hospital, Central South University, Hunan 410078, China; [2]NHC Key Laboratory of Carcinogenesis (Central South University), Cancer Research Institute and School of Basic Medicine, Central South University, Changsha, Hunan 410078, China; [3]Shenzhen Institute of Pediatrics, Shenzhen Children's Hospital, 7019 Yi Tian Road, Shenzhen 518038, China; [4]Division of Hematology and Oncology, Shenzhen Children's Hospital, 7019 Yi Tian Road, Shenzhen 518038, China; [5]Department of Biomedical and Molecular Sciences, Queen's University, Kingston, Ontario, Canada; [6]Department of Pathology, School of Basic Medicine and Xiangya Hospital, Central South University, Changsha, Hunan 410008, China; [7]Department of Oncology, Institute of Medical Sciences, Xiangya Hospital, Central South University, Changsha, Hunan 410008, China; [8]Hunan Key Laboratory of Tumor Models and Individualized Medicine, Department of Thoracic Surgery, Second Xiangya Hospital, Central South University, Changsha 410011, China

Ferroptosis is primarily caused by intracellular iron catalytic activity and lipid peroxidation. The potential interplay between ferroptosis and apoptosis remains poorly understood. Here, we show that the expression of a nuclear long non-coding RNA (lncRNA), LINC00618, is reduced in human leukemia and strongly increased by vincristine (VCR) treatment. Furthermore, LINC00618 promotes apoptosis by increasing the levels of BCL2-Associated X (BAX) and cleavage of caspase-3. LINC00618 also accelerates ferroptosis by increasing the levels of lipid reactive oxygen species (ROS) and iron, two surrogate markers of ferroptosis, and decreasing the expression of solute carrier family 7 member 11 (SLC7A11). Interestingly, VCR-induced ferroptosis and apoptosis are promoted by LINC00618, and LINC00618 accelerates ferroptosis in a manner dependent upon apoptosis. LINC00618 attenuates the expression of lymphoid-specific helicase (LSH), and LSH enhances the transcription of SLC7A11 after the recruitment to the promoter regions of SLC7A11, further inhibiting ferroptosis. Knowledge of these mechanisms demonstrates that lncRNAs related to ferroptosis and apoptosis are critical to leukemogenesis and chemotherapy.

INTRODUCTION

Ferroptosis, a unique, nonapoptotic modality of cell death, is mainly caused by intracellular iron catalytic activity and lipid peroxidation, characterized by the accumulation of reactive oxygen species (ROS).[1] Mechanistically, increased expression of the glutamate-cystine antiporter system x_c^- inhibits the ferroptosis of cancer cells and is negatively correlated with patient outcome. As a unit of system x_c^-, emerging evidence supports that the solute carrier family 7 member 11 (SLC7A11/xCT) is highly expressed in several tumor cells and attenuates erastin-induced ferroptosis.[2–4] An epigenetic decrease of histone H2A ubiquitination represses SLC7A11 expression and decreases occupancy on the promoter of SLC7A11 that facilitates ferroptotic processes.[5,6] Furthermore, the occupancy of histone H2B monoubiquitination (H2Bub1) on the SLC7A11 gene regulatory region is epigenetically reduced and accelerates the occurrence of ferroptosis.[7] Theoretical studies regarding epigenetic modifications of SLC7A11 participating in ferroptosis and cancer therapy are warranted.

Long non-coding RNAs (lncRNAs), with or without a limit protein-encoding capability, are strictly regulated. lncRNAs are involved in multiple regulatory processes, such as chromatin kinetics, RNA processing, and protein synthesis, and function in the metabolism, development, and survival of cancer cells.[8,9] Although lncRNAs may directly attach to transcriptional regulation genes, a quantity of lncRNAs exhibits epigenetic roles on cell growth, apoptosis, and ferroptosis by influencing the post-transcriptional modifications and stability of proteins, like c-Myc and p53.[10–13] However, there is no evidence that lncRNAs are associated with ferroptosis in tumors other than lung cancer.[12,13] Furthermore, a few lncRNAs, including lncRNA HOTAIR and lncRNA H19, have been explored on their functions in leukemia relative to solid tumors.[14] Hence, the matter of how the lncRNAs mediate leukemia cell ferroptosis and tumorigenesis is urgently required.

Lymphoid-specific helicase (LSH/HELLS), a chromatin-remodeling ATPase complex, belongs to the sucrose non-fermentable 2 (SNF2)

Received 14 May 2020; accepted 15 September 2020;
https://doi.org/10.1016/j.ymthe.2020.09.024.

[9]These authors contributed equally to this work.

Correspondence: Yongguang Tao, Department of Pathology, Xiangya Hospital, Central South University, Hunan 410078, China.
E-mail: taoyong@csu.edu.cn
Correspondence: Feiqiu Wen, Shenzhen Institute of Pediatrics, Shenzhen Children's Hospital, 7019 Yi Tian Road, Shenzhen 518038, China.
E-mail: fwen62@126.com

This is an open access article under the CC BY license (http://creativecommons.org/licenses/by/4.0/).

family and plays a vital role in the normal life processes of animals, plants, and cancer progression.[13,15–23] Interestingly, the lncRNA-LSH interaction exhibits an increased epigenetic regulatory role in ferroptosis and apoptosis in lung cancer cells by dominating the transcriptional process of several metabolic genes.[13,17] It is suggested that the correlation between lncRNAs and LSH may have a favorable effect on leukemogenesis jointly.

Ferroptotic cell death is morphologically and genetically distinct from, but seems to be linked to, apoptosis. For example, induction of ferroptosis through epigenetic repression of bromodomain-containing protein 4 (BRD4) can also efficiently increase apoptosis.[24] Whereas Bcl-2 can be suppressed by ferroptosis inhibitors, the implication that the anti-apoptotic protein Bcl-2 is involved in ferroptosis requires further investigation.[25] Moreover, inactivating glutathione peroxidase 4 (GPX4) does not only accelerate the progression of ferroptosis but also sensitizes cells to apoptosis and necroptosis.[26] As a critical inhibitor of SLC7A11, erastin precipitates iron, which leads to subsequent lipid peroxidation. The increase of iron levels promotes ROS production, which contributes to subsequent ferroptotic and apoptotic cell death and inhibits the progression of proliferation and differentiation.[27–29] In addition, the erastin-inhibited cystine/glutamate antiporter confers cisplatin-resistant head and neck cancer cell sensitivity.[30] Overall, this suggests a possible link between apoptotic inducers and ferroptosis. Although ferroptosis may be related to programmed cell deaths, like apoptosis, necroptosis, etc.,[31] the detailed mechanism remains elusive. In this study, we investigate the epigenetic regulatory role of the novel lncRNA, LINC00618, in ferroptosis and apoptosis in leukemia cells.

RESULTS

LINC00618 Is Induced by VCR and Highly Downregulated in Human Acute Myeloid Leukemia

To choose sensitive anti-tumor reagents effectively, three leukemia cells, K562, HL60, and MV4-11, were treated with four chemotherapeutic reagents, including vincristine (VCR) (Figure 1A) and 6-mercatopurine monohydrate (6MP), imatinib mesylate (IM), and AZD9496 (AZD) (Figures S1A–S1C) at the indicated doses, respectively. Notably, since their growth is limited to a low dose of VCR, we mainly focused on VCR as candidates in leukemia research. Moreover, VCR is widely used in the treatment of leukemia, brain tumors, and solid tumors and exhibits powerful anti-cancer effects in both adults and children. It mainly inhibits the polymerization of tubulin dimer and mitosis, thus inducing cell apoptosis.[32,33]

To select several differentially expressed lncRNAs related to metabolic processes, we analyzed and identified target lncRNAs through our previous RNA-sequencing data from lung cells.[17] Subsequently, according to the data from The Cancer Genome Atlas (TCGA)-LAML, we screened the expression profiles of top-ranked lncRNAs from lung cancer and analyzed their expression level in acute myeloid leukemia (AML) (Figure 1B). Combined with differentially expressed lncRNAs derived from our RNA-sequencing data, we focused on long, intragenic non-protein coding RNA 00618 (LINC00618) as the top-ranked candidate in leukemia cells. To define if LINC00618 could be influenced by VCR, quantitative reverse transcription PCR (qRT-PCR) assays indicated that a time-dependent increase of LINC00618 was observed both in MV4-11 cells and K562 cells using VCR treatment for 24 h and 48 h (Figure 1C), suggesting that LINC00618 might serve as a chemotherapeutic marker.[34] To evaluate the clinical significance of LINC00618 in cancer progression, we first collected 28 AML patient samples and 10 normal samples and systematically assessed the relative LINC00618 expression in cancer. It was found to exhibit a significant downregulation of LINC00618 in AML (Figure 1D). To explore the location of LINC00618, we used Cy3-labeled LINC00618 probes and performed a single molecule RNA fluorescence *in situ* hybridization (RNA FISH) experiment (Figure 1E), which indicated that LINC00618 was primarily located in the nucleus of MV4-11 cells, K562 cells, and HL60 cells. Moreover, the nuclear and cytoplasmic RNA fractions experiment also supported the nuclear localization of LINC00618 (Figure S2A). These findings suggest that LINC00618 displays critical biological functions in the cellular nucleus and may serve as a useful mediator in leukemia cells.

Overexpression of LINC00618 Promotes Cell Apoptosis and Ferroptosis

It is shown that the expression level of LINC00618 in HL60 cells was higher than that in K562 cells and MV4-11 cells (Figure S2B). To uncover the biological effects of LINC00618, we stably overexpressed LINC00618 in K562 and MV4-11 cell lines by a lentiviral vector. Subsequently, the efficiency of overexpression was detected by qRT-PCR (Figures 2A and 2B). After that, to investigate the role of LINC00618 on apoptosis, cell viability assays illustrated that overexpression of LINC00618 significantly conferred cell lines' sensitivity to VCR (Figures 2C and 2D). Moreover, we found that LINC00618 induced a greater level of apoptosis, as measured by Annexin V and phycoerythrin (PE) staining in K562 and MV4-11 cells maintaining LINC00618 overexpression (Figures S3A and S3B). Stable overexpression of LINC00618 in K562 and MV4-11 cells decreased the level of full-length caspase-3 protein and increased the levels of cleaved caspase-3 and BCL2-Associated X (BAX) protein dramatically (Figure 2E), further demonstrating that LINC00618 could promote apoptosis.

Our previous studies indicated that these selected lncRNAs function in apoptosis and ferroptosis caused by intracellular lipid ROS and iron.[12] To address the role of LINC00618 in ferroptosis, we first performed a transmission electron microscopy experiment on LINC00618-overexpressed K562 cells (Figure 2F). As a result, the overexpression of LINC00618 has a slight impact on mitochondrial morphology in K562 cells treated with DMSO. When the two cells were treated with erastin, the morphological features of mitochondria involved a smaller size, increased membrane density, and cristae thickening. Moreover, LINC00618 made mitochondria smaller and increased membrane density in erastin-treated cells. Subsequently, we discovered that LINC00618 could increase both the intracellular levels of ROS in MV4-11 cells (Figure 2G) and in K562 cells (Figure S3C) using probe dihydroethidium (DHE)[35] and the levels of lipid

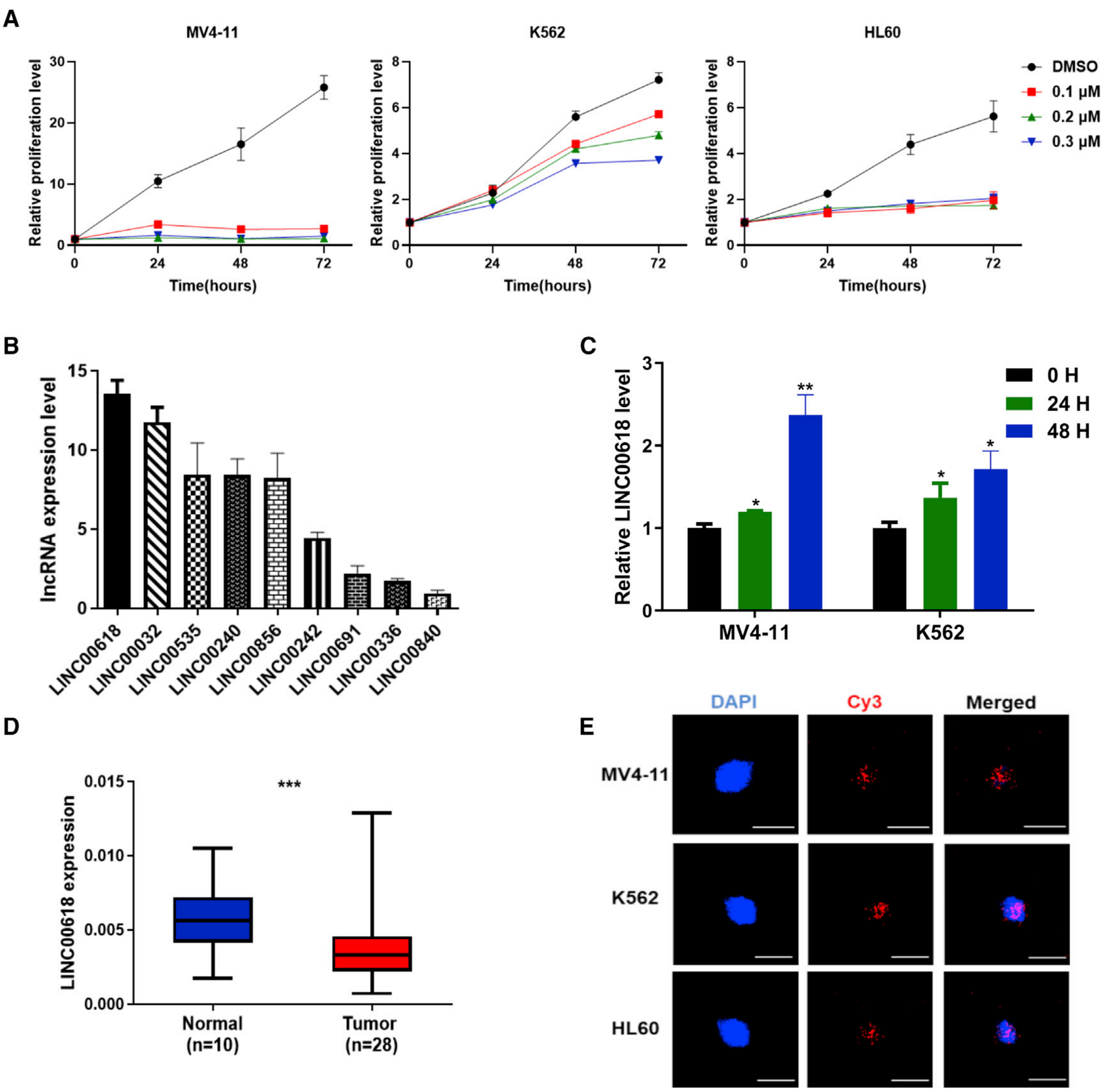

Figure 1. LINC00618 Is Induced by VCR and Highly Downregulated in Human Acute Myeloid Leukemia (AML)

(A) The relative proliferation levels of K562, HL60, and MV4-11 cells checked by MTS assays with the indicated doses of VCR treatment. DMSO served as control. (B) The expression profiling of screened lncRNAs in AML from TCGA-LAML. (C) VCR induced the expression of LINC00618 for 0 h (0 H), 24 h (24 H), and 48 h (48 H) in MV4-11 cells and K562 cells. (D) LINC00618 expression is presented as box plot diagrams in AML patient samples. (E) RNA FISH analysis of LINC00618 localization in MV4-11 cells, K562 cells, and HL60 cells using Cy3-labeled probes. All scale bars, 25 μm long. Data are shown as the mean ± SEM; n ≥ 3 independent experiments, two-tailed Student's t test: ns, nonsignificant ($p > 0.05$), *$p < 0.05$, **$p < 0.01$, ***$p < 0.001$.

ROS (Figures 2H and 2I) using probe C11 BODIPY 581/591[36] after fluorescence-activating cell sorter (FACS) in MV4-11 and K562 cells. Furthermore, overexpression of LINC00618 also increased the concentrations of intracellular iron determined by an iron assay kit (Figures 2J and 2K). Taken together, these results suggested that LINC00618 promotes cell apoptosis and ferroptosis.

Knockdown of LINC00618 Inhibits Cell Apoptosis and Ferroptosis

To further elucidate the physiological role of LINC00618 in leukemia, we performed the stable knockdown of LINC00618 in HL60 cells (Figure 3A) and K562 cells (Figure S3D) with two separate short hairpin RNAs (shRNAs). Subsequent qRT-PCR analysis indicated that the RNA levels of LINC00618 were evidently reduced. Consistent with previous experiments that LINC00618-promoted apoptosis, knockdown of LINC00618 dramatically enhanced the cellular resistance to VCR (Figure 3B). Moreover, knockdown of LINC00618 increased the levels of full-length caspase-3 and decreased caspase-3 splicer and BAX in HL60 cells (Figure 3C). In addition, we found that LINC00618 repression reduced the level of early apoptosis as measured by Annexin V and phosphatidylinositol (PI) staining (Figures 3D and 3E). Therefore, the role of LINC00618 in apoptotic promotion was firmly confirmed.

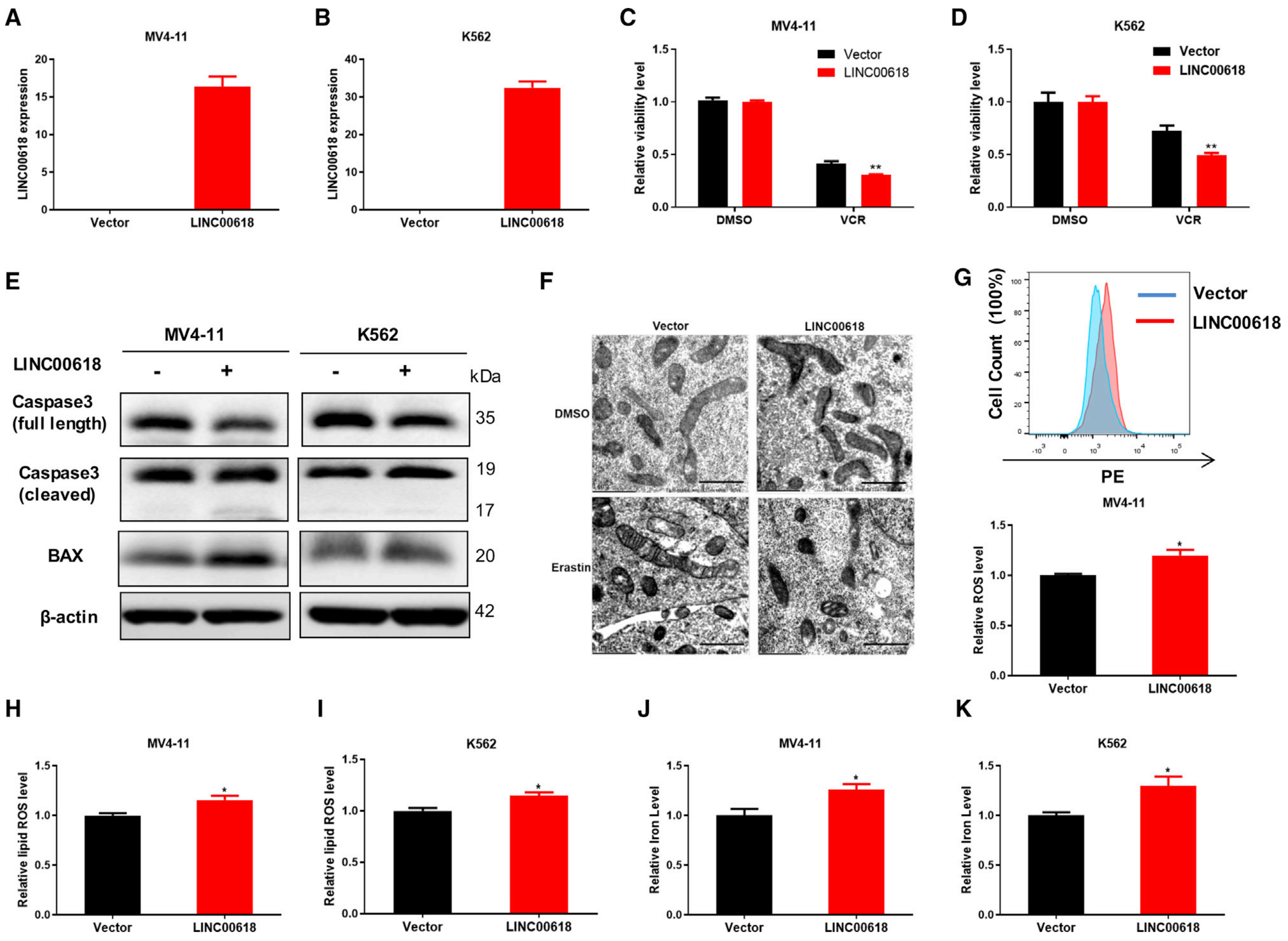

Figure 2. Overexpression of LINC00618 Promotes Cell Apoptosis and Ferroptosis

(A) qRT-PCR analyses of stably expressing vector and LINC00618 in MV4-11 cells. (B) qRT-PCR analyses of stably expressing vector and LINC00618 in K562 cells. (C) Statistical analysis of the relative cell viability in MV4-11 cells stably overexpressing LINC00618 and treated by VCR at 48 h. DMSO served as control. (D) Statistical analysis of cell viability in K562 cells stably overexpressing LINC00618 and treated by VCR at 48 h. DMSO served as control. (E) Western blot analyses of caspase-3 and BAX protein level in K562 and MV4-11 cells stably overexpressing LINC00618. (F) Electron microscopy images of mitochondria in LINC00618-overexpressed K562 cell lines. Scale bars, 1 μm. (G) FACS and statistical analysis of ROS level in MV4-11 cells stably overexpressing LINC00618. (H) FACS and statistical analysis of relative lipid ROS level in MV4-11 cells stably overexpressing LINC00618. (I) FACS and statistical analysis of relative lipid ROS level in K562 cells stably overexpressing LINC00618. (J) Relative iron level checked by an Iron Assay Kit in MV4-11 cells stably overexpressing LINC00618. (K) Relative iron level checked by an Iron Assay Kit in K562 cells stably overexpressing LINC00618. Data are shown as the mean ± SEM; n ≥ 3 independent experiments, two-tailed Student's t test: ns, nonsignificant ($p > 0.05$), *$p < 0.05$, **$p < 0.01$, ***$p < 0.001$.

Subsequently, we explored the role of LINC00618 knockdown in ferroptosis. We observed that the intracellular levels of ROS in HL60 cells (Figure 3F) and in K562 cells (Figure S3E) and lipid ROS (Figure 3G) were decreased along with knockdown of LINC00618 in HL60 cells. Moreover, a repressed LINC00618 decreased the concentration of iron in cells (Figure 3H). Together, our data illustrated that LINC00618 not only accelerated cell apoptosis but also promoted ferroptosis.

LINC00618 Accelerates Ferroptosis in Dependence of Apoptosis

Ferroptosis does not share characteristics with apoptosis. Ferroptosis protects cells from oxidative stress rather than activating cell death machinery-like apoptosis.[31] To investigate the potential relationship between apoptosis and ferroptosis, we first treated the LINC00618 overexpression of K562 cell lines with VCR and detected their apoptosis levels, as measured by Annexin V and PI staining. It was found that LINC00618 increased cellular sensitivity and promoted VCR-induced cell death, which was blocked by caspase inhibitor Z-VAD-FMK (VAD). However, when VCR and erastin were added into these cells, the VCR and erastin-treated group did not show a significant effect relative to VCR-induced apoptosis (Figure 4A). These findings demonstrate that LINC00618 augmented apoptosis, and ferroptosis activators did not change the apoptosis levels.

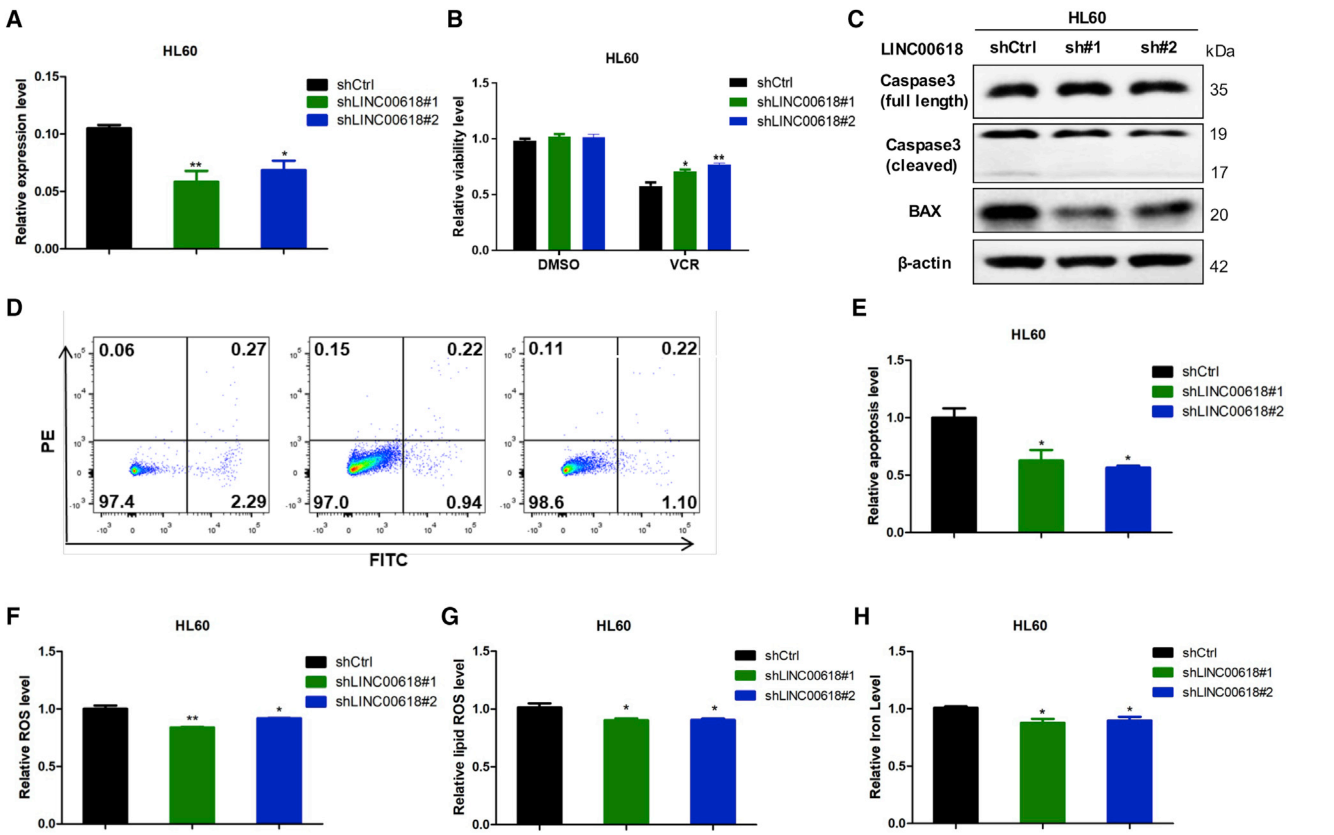

Figure 3. Knockdown of LINC00618 Inhibits Cell Apoptosis and Ferroptosis

(A) qRT-PCR analyses of stably knockdown vector and LINC00618 in HL60 cells. (B) Statistical analysis of cell viability in HL60 cells stably overexpressing LINC00618 and treated by VCR at 48 h. DMSO served as control. (C) Western blot analyses of caspase-3 and BAX protein levels in HL60 cells after the knockdown of LINC00618. (D and E) FACS (D) and statistical analysis (E) showed that LINC00618 promoted early apoptosis in HL60 cells after the knockdown of LINC00618. (F) FACS and statistical analysis of relative ROS level in HL60 cells after the knockdown of LINC00618. (G) FACS and statistical analysis of relative lipid ROS level in HL60 cells after the knockdown of LINC00618. (H) Relative iron level checked by an Iron Assay Kit in HL60 cells after the knockdown of LINC00618. Data are shown as the mean ± SEM; $n \geq 3$ independent experiments, two-tailed Student's t test: ns, nonsignificant ($p > 0.05$), *$p < 0.05$, **$p < 0.01$, ***$p < 0.001$.

Since erastin could not induce apoptosis, we performed the same treatment on K562 cells stably overexpressing LINC00618 to detect the change level of ferroptosis induced by VCR. It was found that LINC00618 dramatically increased the levels of lipid ROS initiated by VCR. After VCR and VAD were used to treat these cells, the lipid ROS levels of overexpressed cells were lower relative to control cells (Figure 4B). However, when VCR and erastin were added into cells, they did not show a significant effect relative to VCR-induced ferroptosis. These results indicated that LINC00618 promoted ferroptosis in dependence of apoptosis and ferroptosis activators did not change VCR-induced ferroptosis.

The correlation between apoptosis and ferroptosis was then further explored. The knockdown of LINC00618 decreased cellular sensitivity and inhibited VCR-induced apoptosis, which was blocked by VAD in HL60 cells. However, when VCR and erastin were added into these cells, they did not show a significant effect relative to VCR-induced apoptosis (Figure 4C). To detect ferroptosis induced by VCR, we found that the knockdown of LINC00618 dramatically decreased the levels of lipid ROS. After VCR and VAD treated these cells, the lipid ROS levels of control cells decreased greatly relative to knockdown cells (Figure 4D). However, when VCR and erastin were added into these cells, they did not show a significant effect relative to VCR-induced ferroptosis. Taken together, these results indicate that ferroptosis activators do not change VCR-induced apoptosis and ferroptosis, and ferroptosis initiated by LINC00618 depends on VCR-initiated apoptosis.

To verify that LINC00618 accelerates ferroptosis dependent on apoptosis, we first performed mitochondrial membrane potential experiments using JC-10 and found that LINC00618 decreased mitochondrial membrane potential in LINC00618-overexpressed K562 cells treated with DMSO. Moreover, LINC00618 significantly decreased mitochondrial membrane potential in the VCR-treated group (Figure S4A). Conversely, LINC00618 repression increased mitochondrial membrane potential in LINC00618-knockdown

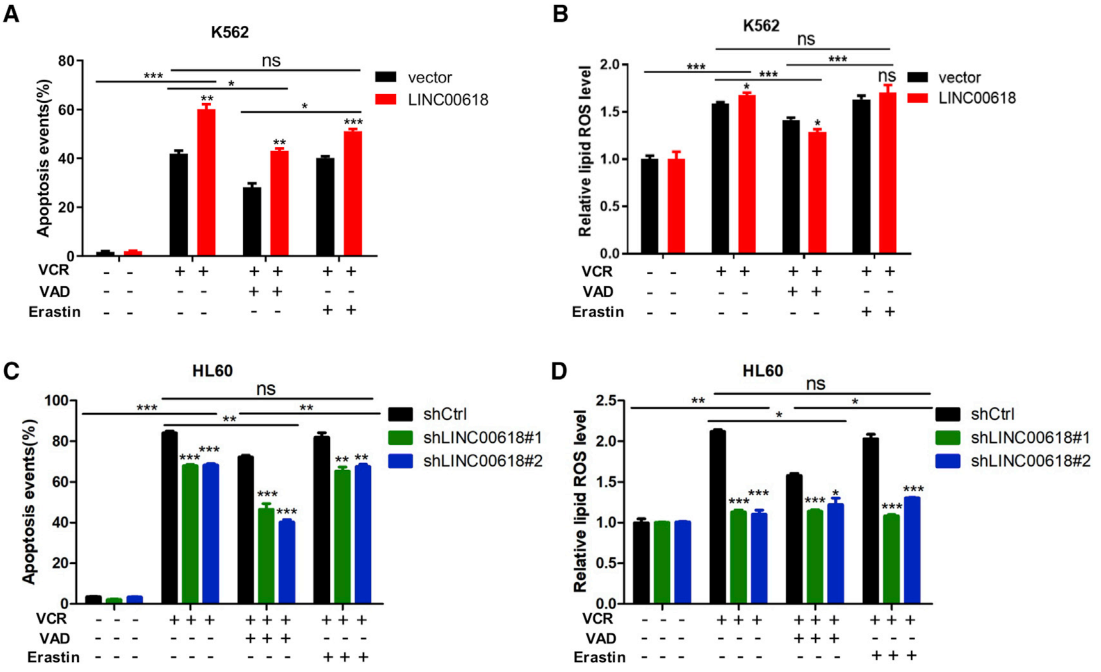

Figure 4. LINC00618 Accelerates Ferroptosis in Dependence of Apoptosis

(A) Flow cytometry and statistical analysis of apoptosis level in K562 cells stably overexpressing LINC00618 with VCR, VAD, and erastin treatment. (B) Flow cytometry and statistical analysis of relative lipid ROS level in K562 cells stably overexpressing LINC00618 with VCR, VAD, and erastin treatment. (C) Flow cytometry and statistical analysis of apoptosis levels in HL60 cells after the knockdown of LINC00618 with VCR, VAD, and erastin treatment. (D) Flow cytometry and statistical analysis of relative lipid ROS levels in HL60 cells after the knockdown of LINC00618 with VCR, VAD, and erastin treatment. Data are shown as the mean ± SEM; $n \geq 3$ independent experiments, two-tailed Student's t test: ns, nonsignificant ($p > 0.05$), *$p < 0.05$, **$p < 0.01$, ***$p < 0.001$.

HL60 cells (Figure S4B). Besides, repressed LINC00618 had a slight effect on mitochondrial membrane potential in LINC00618-knockdown K562 cells treated with DMSO. Repressed LINC00618 dramatically increased mitochondrial membrane potential in the VCR-treated group (Figure S4C).

Next, overexpression of LINC00618 slightly increased the levels of cleaved caspase-3 in K562 and MV4-11 cells. After VCR exposure, LINC00618 significantly decreased the levels of full-length caspase-3 and increased the levels of cleaved caspase-3 (Figure S5A). Conversely, cleaved caspase-3 was decreased in HL60 cells stably interfering with LINC00618 after VCR exposure (Figure S5B), further demonstrating that LINC00618 promotes cell apoptosis.

SLC7A11 Is Regulated by LINC00618 through LSH

Next, to elucidate the concrete mechanisms of the effects of LINC00618 on ferroptosis, the ferroptosis-associated genes were analyzed and screened using qPCR arrays in K562 cells stably overexpressing LINC00618 (Figure 5A).[17,37] Among these candidates, we focused on the differentially expressed SLC7A11 as top-ranked genes. Based on our previous study, it is indicated that the lncRNA-LSH interaction can perform functions in ferroptosis by decreasing intracellular iron concentrations and lipid ROS levels.[13] We hypothesized that LINC0618 could interact with LSH, affecting SLC7A11 expression, inducing in ferroptosis.

First, overexpression of LINC00618 did not affect the mRNA levels of LSH in K562 cells and had little influence on the mRNA levels of LSH in MV4-11 cells (Figures S6A and S6B). However, western blot indicated that the levels of both LSH and SLC7A11 were decreased in K562 and MV4-11 cells stably overexpressing LINC00618, and ferroptosis suppressor GPX4 was also decreased, and the ferroptosis promoters, including acyl-coenzyme A synthetase long-chain family member 4 (ACSL4) and cyclooxygenase 2 (COX2), were increased by LINC00618 overexpression (Figure 5B). Conversely, LSH, SLC7A11, and GPX4 were increased in HL60 cells after the knockdown of LINC00618, and ACSL4 and COX2 were decreased by LINC00618 repression (Figure 5C). Thus, the role of LINC00618 in promoting ferroptosis was further demonstrated by ferroptosis markers.

To determine the correlation of LSH and SLC7A11, we observed that LSH might have a positive correlation with SLC7A11 from the four parent cells (Figure 5D). First, according to TCGA-LAML samples (Figure 5E) and the clinical resources of AML (Figure 5F), we found that there was a positive correlation between them. To further demonstrate whether LSH could upregulate SLC7A11 or not, we

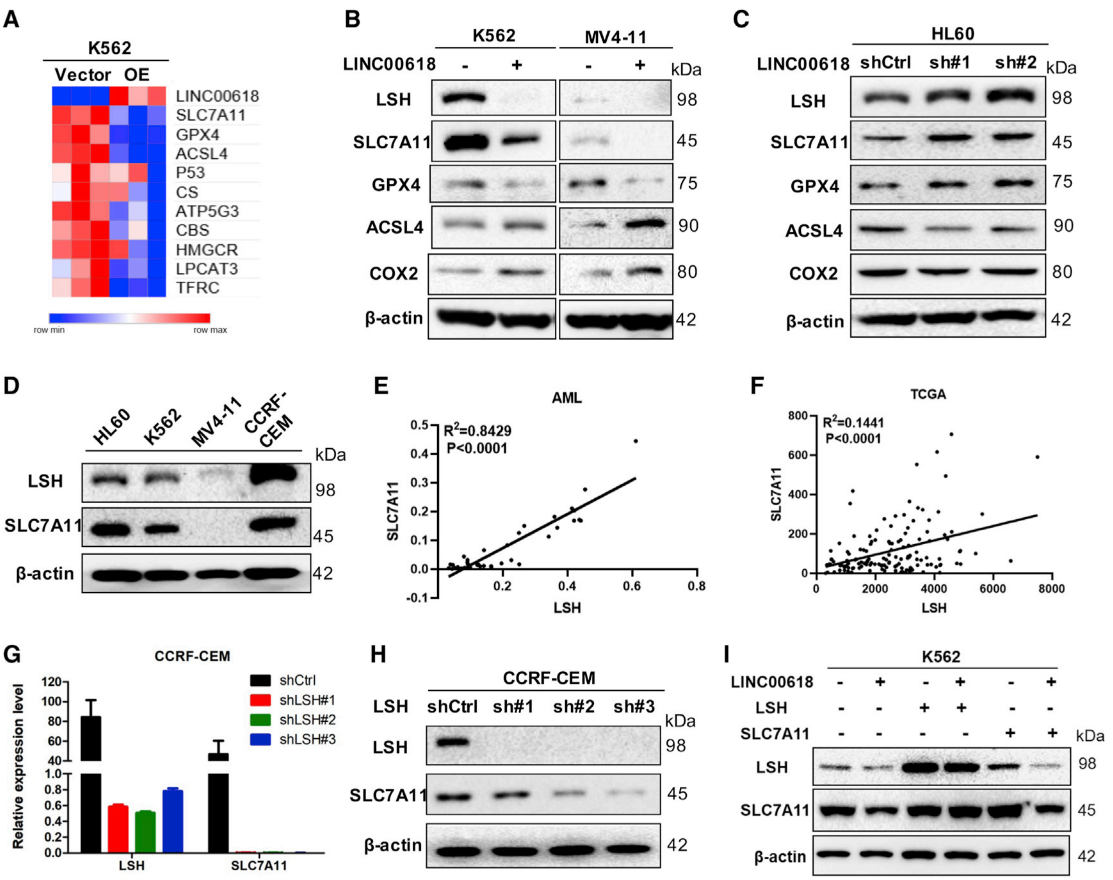

Figure 5. SLC7A11 Is Regulated by LINC00618 through LSH

(A) qPCR-based heatmap indicating the changes of ferroptosis-related genes in K562 cells stably overexpressing LINC00618. (B) Western blot analyses of LSH, SLC7A11, and ferroptosis-related proteins in K562 and MV4-11 cells stably overexpressing LINC00618. (C) Western blot analyses of LSH, SLC7A11, and ferroptosis-related proteins in HL60 cells after the knockdown of LINC00618. (D) Protein expression level of LSH and SLC7A11 in four leukemia cells. (E) The correlation between LSH and SLC7A11 mRNA level was measured in 38 AML tissues. (F) The correlation between LSH and SLC7A11 mRNA level was analyzed in TCGA-LAML samples. (G) qPCR analyses of SLC7A11 in CCRF-CEM cells after the knockdown of LSH. (H) Western blot analyses of SLC7A11 in CCRF-CEM cells after the knockdown of LSH. (I) Western blot analyses of LSH and SLC7A11 in K562 cells stably overexpressing LINC00618 with LSH or SLC7A11 overexpression. Data are shown as the mean ± SEM; n ≥ 3 independent experiments, two-tailed Student's t test: ns, nonsignificant ($p > 0.05$), *$p < 0.05$, **$p < 0.01$, ***$p < 0.001$.

found that both the mRNA levels and protein levels of SLC7A11 were decreased by knockdown of LSH in CCRF-CEM cells (Figures 5G and 5H). Therefore, we hypothesized that LSH might bind to the promoter regions of SLC7A11 and positively regulate SLC7A11, which was regulated by LINC00618.

To further illustrate the relationship among LINC00618, LSH, and SLC7A11, we observed that LINC00618 overexpression decreased the protein levels of LSH and SLC7A11, and LSH overexpression increased the protein levels of SLC7A11 in both K562 cells (Figures 5I, S6C, and S6D) and MV4-11 cells (Figure S6E), stably overexpressing LINC00618, whereas SLC7A11 overexpression did not affect LSH expression. Simultaneously, with the use of these transfected cells, it was proven that LINC00618 overexpression increased cellular sensitivity to erastin, whereas LSH or SLC7A11 overexpression could up-regulate SLC7A11 levels and resist cellular ferroptosis in response to erastin. However, the transfected cells overexpressing LINC00618 increased cellular sensitivity to VCR, whereas the transfected cells overexpressing LSH or SLC7A11 had no ability to resist apoptosis when treated with VCR (Figure S6D). Furthermore, SLC7A11 overexpression did not affect LSH expression in CCRF-CEM cells after the knockdown of LSH (Figure S6F). This indicates that LINC00618 inhibited SLC7A11 expression by LSH, accelerating ferroptosis in dependence of apoptosis.

LINC00618 Recruits LSH to the Promoter Regions of SLC7A11

Our previous study has shown that lncRNA interacts with LSH and influences LSH binding to the promoter of target genes. The interaction is strongly correlated with lung cancer progression and poor survival.[38] To validate the interaction between LINC00618 and certain proteins of LSH, RNA immunoprecipitation (RIP) experiments were conducted to confirm that an enrichment of LINC00618 could

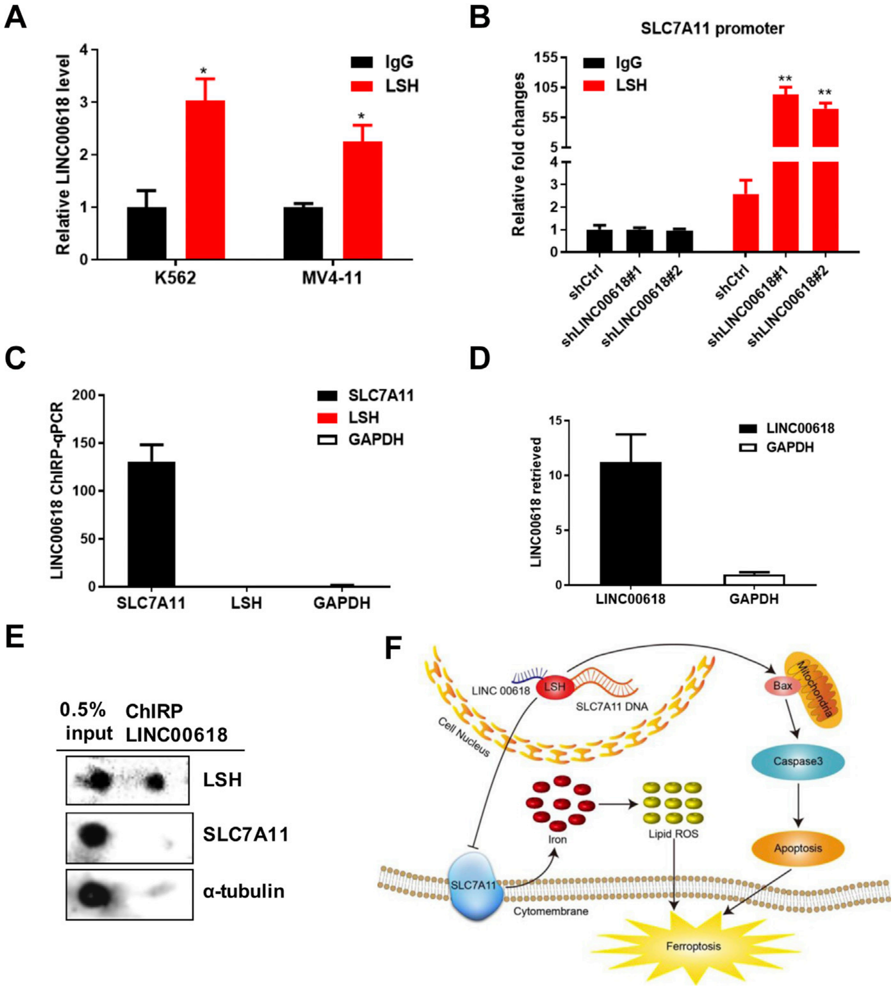

Figure 6. LINC00618 Recruits LSH to the Promoter Regions of SLC7A11

(A) RIP assays indicated that LINC00618 can be sequestered by LSH in K562 and MV4-11 cells. (B) ChIP-qPCR assays indicate that LSH is recruited to the promoter regions of SLC7A11 in HL60 cells after the knockdown of LINC00618 with SLC7A11 ChIP primer #1. (C) ChIRP enriches for LINC00618. GAPDH served as negative controls. (D) LINC00618 ChIRP-qPCR detects LSH and SLC7A11. (E) Dot blot analyses of LINC00618 ChIRP retrieves LSH and SLC7A11. (F) Working model for LINC00618 in cell apoptosis and ferroptosis. Nuclear LINC00618 amplification reduces LSH expression. Decreased LSH inhibits SLC7A11 transcription and expression through occupying the promoter, leading to cell ferroptosis. Moreover, LINC00618 overexpression increases the expression levels of BAX and cleaved caspase-3 and in turn, triggers apoptosis. Lastly, LINC00618 accelerates ferroptosis independent of apoptosis. Data are shown as the mean ± SEM; $n \geq 3$ independent experiments, two-tailed Student's t test: ns, nonsignificant ($p > 0.05$), *$p < 0.05$, **$p < 0.01$, ***$p < 0.001$.

be precipitated with the antibody against LSH in MV4-11 and K562 cells (Figure 6A). LSH always plays a significant role in binding to the promoters of target genes and affecting their transcription.[16,39] To determine whether LSH could bind to the promoter regions of SLC7A11, we predicted the promoter binding sites of SLC7A11 through the University of California, Santa Cruz (UCSC), Genome Browser and carried out chromatin immunoprecipitation (ChIP) experiments. We observed that the enrichment of LSH to SLC7A11 promoter regions was increased significantly in HL60 cells after the knockdown of LINC00618 (Figure 6B), indicating that LINC00618 could regulate SLC7A11 through LSH. Chromatin isolation by RNA purification (ChIRP) experiments firmly established that LINC00618 RNA was specifically retrieved, whereas glyceraldehyde 3-phosphate dehydrogenase (GAPDH) served as a negative control using biotin-labeled LINC00618 probes in HL60 cells[40] (Figure 6C). Subsequently, qRT-PCR showed that LINC00618 probes yielded comparable enrichment of SLC7A11 DNA regions over negative regions of GAPDH and LSH DNA regions (Figure 6D). Combined with dot blot assays, we found that LINC00618 ChIRP could specifically retrieve LSH proteins, whereas SLC7A11 and tubulin were not detected (Figure 6E).

Collectively, we have characterized LINC00618 as a potential tumor suppressor in leukemia tumorigenesis. Based on our findings, we

propose a model that LINC00618 accelerates ferroptosis dependent on apoptosis (Figure 6F). LINC00618 attenuates the expression of LSH, whereas LSH enhances the transcription of SLC7A11 after the recruitment to the promoter regions of SLC7A11, further inhibiting ferroptosis. Moreover, LINC00618 increases the expression of BAX and cleaved caspase-3 and in turn, triggers apoptosis.

DISCUSSION

Drug resistance and disease recurrence are the major obstacles that need to be urgently eliminated in chemotherapy of AML. Although VCR is used to abate tumors extensively, prolonged use of VCR increases cancer cells' resistance to it. The abnormal expression of multiple upregulated and downregulated lncRNAs is critical to VCR resistance in colon cancer cells. Therefore, lncRNAs associated with VCR are increasingly regarded as key regulators with the potential to improve therapeutic sensitivity to chemotherapy.[41–43] Besides, studies have reported that lncRNAs, including MRUL and LINC00518, increase cellular sensitivity to multiple drugs, like VCR and Adriamycin (ADR), by affecting the expression of multidrug-resistant proteins.[44,45] However, the research of lncRNA involvement in VCR-treated leukemia has not yet been explored. Here, we have found that VCR-related lncRNA LINC00618 is highly expressed in leukemia cells and is located in the cell nucleus, suggesting that it plays a critical role in leukemia chemotherapy.

It is found that a subset of lncRNAs could serve as an independent mediator for diagnosis, treatment, and prognosis in AML.[46,47] Moreover, lncRNAs are closely associated with apoptosis and exert a critical epigenetic regulatory role in leukemogenesis.[48,49] However, there is no evidence that lncRNA functions in ferroptosis in cancers other than lung cancer.[13] Here, our results indicate that LINC00618 is highly downregulated in AML and can promote the progression of both cell apoptosis and ferroptosis in leukemia cells. Emerging evidence that ferroptosis is related to chemotherapeutic resistance indicates that there is a close interconnection between apoptosis and ferroptosis and their molecular mechanisms. For example, ferroptosis is an indispensable process of acute injury induced by cisplatin.[25,50,51] Inhibition of ferroptosis by key regulators may induce apoptosis of chemotherapy-resistant cancer cells.[30] Studies have revealed that oxidative stress-induced ferroptosis cooperating with apoptotic inhibition promotes long-term cellular well-being.[52] Recently, ferroptosis suppressor protein 1 (FSP1), which was known as an apoptosis-inducing factor previously, synergizes with GPX4 inhibitors to trigger ferroptosis.[53,54] However, the detailed relation that lncRNAs link apoptosis and ferroptosis remains unknown. Since LINC00618 plays a role in apoptosis and ferroptosis, we treat cells with VCR to check the apoptosis and ferroptosis levels. Unexpectedly, overexpression of LINC00618 promotes apoptosis and ferroptosis markedly in K562 cells after VCR exposure. However, VCR and erastin treatment does not further affect apoptosis and ferroptosis levels relative to VCR treatment in K562 cells (Figures 4A and 4B). Our results demonstrate that ferroptosis depends on apoptosis. However, the reason why LINC00618 is able to link ferroptosis to apoptosis requires further investigation.

Generally, lncRNAs perform their function by interacting with proteins. In particular, lncRNAs interacting with SWItch (SWI)/SNF chromatin-remodeling complexes have widely drawn our attention to the development of various cancers. lncRNAs may be capable of binding or recruiting subunits of SWI/SNF complexes to modulate the expression of multiple target genes.[55,56] To explore the concrete mechanism of ferroptosis, we focus on the SLC7A11 through the differential expression of VCR-induced genes. In our study, we illustrate that LINC00618 decreases the expression of SLC7A11 through interacting with LSH, thus inhibiting ferroptosis. Moreover, the overexpression of LINC00618 increases the expression level of BAX and cleaved caspase-3 and promotes apoptosis. Importantly, LINC00618 accelerates ferroptosis in dependence of apoptosis. Thus, we do not rule out the possibility that different manners of cell death have a close connection.

MATERIALS AND METHODS

Cell Culture, Chemicals, Plasmids, and Small Interfering RNAs (siRNAs)

Leukemia cell lines HL60 and K562 were obtained from the Cancer Research Institute of Central South University. MV4-11 and CCRF-CEM were obtained from American Type Culture Collection (ATCC). All leukemia cells were cultured in RPMI-1640 media (Gibco, USA), supplemented with 10% (v/v) fetal bovine serum (FBS). 293T cells were cultured in DMEM medium (Gibco, USA), supplemented with 10% (v/v) FBS. All cell lines were maintained at 37°C with 5% CO_2. VCR, VAD, and erastin were purchased from Selleck. LSH, LINC00618, and SLC7A11 cDNA clones were purchased from Vigene Biosciences. Sequences for LINC00618 shRNA number (#)1 and shRNA #2 are as follows: 5′-GCAGTTTCCAGAGATGAATTT-3′, 5′-GTCCTTGCAAGTAGGTAAATG-3′. The transfection of plasmids was performed using LipoMax (Sudgen), and stably expressing colonies were selected using 1 mg/mL puromycin.

Quantitative Real-Time PCR and Leukemia Patient Samples

The details of these procedures have been described previously. Primers are listed in Table S1. Leukemia patient samples were obtained from Shenzhen Children's Hospital. Written, informed consent was obtained from all patients or their relatives, and the Institutional Ethical Committee of our hospital approved the study in accordance with the ethical guidelines from the Declaration of Helsinki.

RNA FISH

Single-molecule RNA FISH was performed as previously described.[57] Probes were designed by the online probe designer at https://www.biosearchtech.com/products/rna-fish/ and were labeled with Cy3. Probes for LINC00618 are as follows: 5′-ATTCATCTCTGGAAACTGCC-3′.

Cell Viability Assays

Cells (1×10^3 cells/well) were seeded in RPMI-1640 medium (100 μL/well) into 96-well plates with five replicate wells. After a

12-h incubation, 20 μL 3-(4,5-dimethylthiazol-2-yl)-5-(3-carboxymethoxyphenyl)-2-(4-sulfophenyl)-2H-tetrazolium, inner salt (MTS) (Promega; G3580) was added to each well and incubated at 37°C, 5% CO_2, for 2 h. Absorption values were measured at 490 nm on a multiple-well plate reader (BioTek) and at 48 h. The cell viability was expressed as A/B, where A was the absorbance value from the experimental group, and B was that from the control cells.

Western Blot Analysis

Cells were harvested and washed twice with 1 × PBS and lysed in immunoprecipitation (IP) lysis buffer on ice for 2 h. After centrifugation at 15,000 × *g*, 4°C for 15 min, a quantity of 50 μg total protein was used for western blot analysis. The protein lysates were resolved on SDS-PAGE gels and transferred onto a polyvinylidene fluoride (PVDF) transmembrane for 2 h. The membrane was incubated at 4°C overnight with a different primary antibody. The next day, after washing three times with PBST, the membrane was incubated at room temperature with corresponding second antibody and exposed by the ChemiDox XRS+ image-forming system. The following antibodies were used: caspase-3 (Cell Signaling Technology; catalog (cat) #14220), BAX (Proteintech; cat #50599-2-Ig), LSH (Santa Cruz Biotechnology; cat #sc-46665), xCT (SLC7A11) (Proteintech; cat #26864-1-AP), GPX4 (ABclonal; cat #A1933), ACSL4 (Santa Cruz Biotechnology; cat #sc-271800), COX2 (ABclonal; cat #A1253), and β-actin (Sigma; cat #A5411).

Transmission Electron Microscopy

LINC00618-overexpressed K562 cells were seeded onto 6 cm plates and were treated with 10 μM erastin for 48 h. Images were acquired using transmission electron microscope (Hitachi; HT7700).

Measurement of Total ROS, Lipid ROS, and Iron

For total ROS determination, cells were harvested and trypsinized into single cells. After washing with 1 × PBS, cells were resuspended in RPMI-1640 medium plus 10% FBS and a 400-μM DHE (Sigma; cat #37291) probe was added. Samples were incubated at 37°C, 5% CO_2, for 15 min and protected from light. The samples were washed twice with preheated PBS and resuspended in 200 μL PBS. More than 10, 000 cells were analyzed by a flow cytometer (Fortessa, BD Biosciences) with a PE channel.

For lipid ROS determination, cells were treated as indicated and harvested and trypsinized into single cells. After washing with 1 × PBS, cells were resuspended in RPMI-1640 medium plus 10% FBS and a 10-μM C11 BODIPY (Thermo Fisher Scientific; cat #D3861) probe was added. Samples were incubated at 37°C, 5% CO_2, for 15 min and protected from light. The samples were washed twice with preheated PBS. More than 20,000 cells were analyzed by a flow cytometer (Fortessa, BD Biosciences) with fluorescein isothiocyanate (FITC) green channel and Texas red channel.

For the iron determination, the total iron and ferrous iron (Fe^{2+}) were measured by the Iron Assay Kit (Sigma-Aldrich; MAK025) in each cell line. 2×10^6 of cells were rapidly homogenized in 4–10 volumes of iron assay buffer. Samples were centrifuged at 13,000 × *g*, 4°C, for 10 min to remove insoluble material. To measure ferric iron, two sets of wells were set up. Then, a 5-μL iron assay buffer was added to each sample to measure ferrous iron in one set of wells, and a 5-μL iron reducer was added to the other set of wells. The level of ferric iron was calculated by subtracting ferrous iron from total iron. The reactions of samples were mixed well using a horizontal shaker and were incubated for 30 min at room temperature and protected from light. Then, the reactions that a 100-μL iron probe was added to each well containing standard or test samples were incubated for 60 min at room temperature and protected from light. Finally, the absorbance was measured at 593 nm.

Assessment of Mitochondrial Membrane Potential

Cells were seeded onto 6-well plates and were treated with VCR for 24 h. Then, mitochondrial membrane potential was measured by the JC-10 kit (4A Biotech; cat #FXP134).

RIP Assays

RIP was generally performed as previously described.[13] A total of 10^7 cells were harvested and resuspended in 2 mL of PBS. The cell lysate was pelleted by centrifugation at 4°C and 500 × *g* for 15 min, resuspended in 1 mL of RIP buffer, split into three fractions (for input, immunoglobulin G [IgG], and IP), and then centrifuged at 4°C and 13,000 rpm for 10 min. Antibodies against normal mouse IgG (Millipore; cat #12-371) were added to the supernatant and incubated overnight at 4°C with gentle rotation. Next, 40 μL protein A/G beads were added, and the mixture was incubated at 4°C for an additional hour. The beads were pelleted at 2,500 rpm for 30 s, washed three times with 500 μL of RIP buffer and one time with PBS, and then resuspended in 1 mL of RNAiso Plus. The coprecipitated RNAs were detected by qRT-PCR for LINC00618.

ChIP and Quantitative Real-Rime PCR

ChIP-qPCR was generally performed as previously described.[13,17,58] 1×10^7 cells were fixed with 1% formaldehyde for 10 min and were stopped with the addition of 1/10 volume 1.25 M glycine for 5 min at room temperature. After dissolution in a cell lysis buffer, the sonication step was performed on a Qsonica sonicator (3 min, 20 s on, 20 s off). 300 μg protein-chromatin complex was combined with antibodies, which were captured with preblocked Dynabeads protein G (Invitrogen) in each example. ChIP DNA was analyzed by qPCR with SYBR Green (Roche) on an ABI-7500 (Applied Biosystems) using the primers specified in Table S2. The antibodies used are as follows: LSH (Santa Cruz Biotechnology; cat #sc-46665) and normal mouse IgG (Millipore; cat #12-371).

ChIRP

ChIRP-qPCR was generally performed as previously described[13]. Antisense DNA affinity probes with BiotinTEG at the 3-prime end for selective retrieval of the RNA target were designed at https://www.biosearchtech.com/products/rna-fish/. 2×10^7 cells were acquired for one ChIRP sample. After crosslink with 1% glutaraldehyde, cell pellets were prepared and further lysed in supplemented lysis

buffer. The cell lysates were sonicated and hybridized with biotinylated probes (100 pmol probe per 1 mL chromatin) and incubated at 37°C for 4 h with shaking. Extract RNA and DNA fractions were quantitated by qRT-PCR from ChIRP samples. ChIRP protein elution was analyzed by dot blot. The antibodies used are as follows: LSH (Santa Cruz Biotechnology; cat #sc-46665) and α-tubulin (Santa Cruz Biotechnology; cat #sc-5286).

Statistical Analyses

All statistical analyses were performed using the GraphPad Prism 8.0 software package and SPSS 22.0 statistical software package (Abbott Laboratories, USA) for Windows. The data are presented as mean ± SEM from multiple individual experiments each performed in triplicates. Student's t tests (two-tailed) were applied to compare differences between two groups: ns is nonsignificant ($p > 0.05$), *$p < 0.05$, **$p < 0.01$, and ***$p < 0.001$.

SUPPLEMENTAL INFORMATION

Supplemental Information can be found online at https://doi.org/10.1016/j.ymthe.2020.09.024.

AUTHOR CONTRIBUTIONS

Y.T., F.W., and Z.W. designed and initiated the study. Z.W. performed experiments. N.L., Y.L., and L.O. developed methodology. X.C., Y.S., D.X., and S.L. contributed to data interpretation. Z.W., Y.T., and Y.S. wrote the paper. This manuscript has been read and approved by all authors.

CONFLICTS OF INTEREST

The authors declare no competing interests.

ACKNOWLEDGMENTS

This manuscript is not under consideration for publication elsewhere. We thank the Shenzhen Public Service Platform of Molecular Medicine in Pediatric Hematology and Oncology very much for the kind suggestions. This work was supported by the National Natural Science Foundation of China (81872285 [to Y.S.], 81672787 [to Y.T.], 81672991 and 81874139 [to S.L.], and 81772496 [to D.X.]) and the National Basic Research Program of China (2015CB553903 [to Y.T.]).

REFERENCES

1. Stockwell, B.R., Friedmann Angeli, J.P., Bayir, H., Bush, A.I., Conrad, M., Dixon, S.J., Fulda, S., Gascón, S., Hatzios, S.K., Kagan, V.E., et al. (2017). Ferroptosis: A Regulated Cell Death Nexus Linking Metabolism, Redox Biology, and Disease. Cell *171*, 273–285.

2. Jiang, L., Kon, N., Li, T., Wang, S.J., Su, T., Hibshoosh, H., Baer, R., and Gu, W. (2015). Ferroptosis as a p53-mediated activity during tumour suppression. Nature *520*, 57–62.

3. Wang, W., Green, M., Choi, J.E., Gijón, M., Kennedy, P.D., Johnson, J.K., Liao, P., Lang, X., Kryczek, I., Sell, A., et al. (2019). $CD8^+$ T cells regulate tumour ferroptosis during cancer immunotherapy. Nature *569*, 270–274.

4. Chen, D., Fan, Z., Rauh, M., Buchfelder, M., Eyupoglu, I.Y., and Savaskan, N. (2017). ATF4 promotes angiogenesis and neuronal cell death and confers ferroptosis in a xCT-dependent manner. Oncogene *36*, 5593–5608.

5. Gentric, G., Kieffer, Y., Mieulet, V., Goundiam, O., Bonneau, C., Nemati, F., Hurbain, I., Raposo, G., Popova, T., Stern, M.-H., et al. (2019). PML-Regulated Mitochondrial Metabolism Enhances Chemosensitivity in Human Ovarian Cancers. Cell Metab. *29*, 156–173.e10.

6. Zhang, Y., Shi, J., Liu, X., Feng, L., Gong, Z., Koppula, P., Sirohi, K., Li, X., Wei, Y., Lee, H., et al. (2018). BAP1 links metabolic regulation of ferroptosis to tumour suppression. Nat. Cell Biol. *20*, 1181–1192.

7. Wang, Y., Yang, L., Zhang, X., Cui, W., Liu, Y., Sun, Q.R., He, Q., Zhao, S., Zhang, G.-A., Wang, Y., and Chen, S. (2019). Epigenetic regulation of ferroptosis by H2B monoubiquitination and p53. EMBO Rep. *20*, e47563.

8. James, A.R., Schroeder, M.P., Neumann, M., Bastian, L., Eckert, C., Gökbuget, N., Tanchez, J.O., Schlee, C., Isaakidis, K., Schwartz, S., et al. (2019). Long non-coding RNAs defining major subtypes of B cell precursor acute lymphoblastic leukemia. J. Hematol. Oncol. *12*, 8.

9. Schwarzer, A., Emmrich, S., Schmidt, F., Beck, D., Ng, M., Reimer, C., Adams, F.F., Grasedieck, S., Witte, D., Käbler, S., et al. (2017). The non-coding RNA landscape of human hematopoiesis and leukemia. Nat. Commun. *8*, 218.

10. Huang, M.D., Chen, W.M., Qi, F.Z., Sun, M., Xu, T.P., Ma, P., and Shu, Y.Q. (2015). Long non-coding RNA TUG1 is up-regulated in hepatocellular carcinoma and promotes cell growth and apoptosis by epigenetically silencing of KLF2. Mol. Cancer *14*, 165.

11. Huarte, M. (2015). The emerging role of lncRNAs in cancer. Nat. Med. *21*, 1253–1261.

12. Wang, M., Mao, C., Ouyang, L., Liu, Y., Lai, W., Liu, N., Shi, Y., Chen, L., Xiao, D., Yu, F., et al. (2019). Long noncoding RNA LINC00336 inhibits ferroptosis in lung cancer by functioning as a competing endogenous RNA. Cell Death Differ. *26*, 2329–2343.

13. Mao, C., Wang, X., Liu, Y., Wang, M., Yan, B., Jiang, Y., Shi, Y., Shen, Y., Liu, X., Lai, W., et al. (2018). A G3BP1-Interacting lncRNA Promotes Ferroptosis and Apoptosis in Cancer via Nuclear Sequestration of p53. Cancer Res. *78*, 3484–3496.

14. Liu, Y., Cheng, Z., Pang, Y., Cui, L., Qian, T., Quan, L., Zhao, H., Shi, J., Ke, X., and Fu, L. (2019). Role of microRNAs, circRNAs and long noncoding RNAs in acute myeloid leukemia. J. Hematol. Oncol. *12*, 51.

15. Jia, J., Shi, Y., Chen, L., Lai, W., Yan, B., Jiang, Y., Xiao, D., Xi, S., Cao, Y., Liu, S., et al. (2017). Decrease in Lymphoid Specific Helicase and 5-hydroxymethylcytosine Is Associated with Metastasis and Genome Instability. Theranostics *7*, 3920–3932.

16. He, X., Yan, B., Liu, S., Jia, J., Lai, W., Xin, X., Tang, C.E., Luo, D., Tan, T., Jiang, Y., et al. (2016). Chromatin Remodeling Factor LSH Drives Cancer Progression by Suppressing the Activity of Fumarate Hydratase. Cancer Res. *76*, 5743–5755.

17. Jiang, Y., Mao, C., Yang, R., Yan, B., Shi, Y., Liu, X., Lai, W., Liu, Y., Wang, X., Xiao, D., et al. (2017). EGLN1/c-Myc Induced Lymphoid-Specific Helicase Inhibits Ferroptosis through Lipid Metabolic Gene Expression Changes. Theranostics *7*, 3293–3305.

18. Tao, Y., Liu, S., Briones, V., Geiman, T.M., and Muegge, K. (2011). Treatment of breast cancer cells with DNA demethylating agents leads to a release of Pol II stalling at genes with DNA-hypermethylated regions upstream of TSS. Nucleic Acids Res. *39*, 9508–9520.

19. Tao, Y., Xi, S., Shan, J., Maunakea, A., Che, A., Briones, V., Lee, E.Y., Geiman, T., Huang, J., Stephens, R., et al. (2011). Lsh, chromatin remodeling family member, modulates genome-wide cytosine methylation patterns at nonrepeat sequences. Proc. Natl. Acad. Sci. USA *108*, 5626–5631.

20. Xiao, D., Huang, J., Pan, Y., Li, H., Fu, C., Mao, C., Cheng, Y., Shi, Y., Chen, L., Jiang, Y., et al. (2017). Chromatin Remodeling Factor LSH is Upregulated by the LRP6-GSK3β-E2F1 Axis Linking Reversely with Survival in Gliomas. Theranostics *7*, 132–143.

21. Yang, R., Liu, N., Chen, L., Jiang, Y., Shi, Y., Mao, C., Liu, Y., Wang, M., Lai, W., Tang, H., et al. (2019). LSH interacts with and stabilizes GINS4 transcript that promotes tumourigenesis in non-small cell lung cancer. J. Exp. Clin. Cancer Res. *38*, 280.

22. Yang, R., Liu, N., Chen, L., Jiang, Y., Shi, Y., Mao, C., Liu, Y., Wang, M., Lai, W., Tang, H., et al. (2019). GIAT4RA functions as a tumor suppressor in non-small cell lung cancer by counteracting Uchl3-mediated deubiquitination of LSH. Oncogene *38*, 7133–7145.

23. Chen, L., Shi, Y., Liu, N., Wang, Z., Yang, R., Yan, B., Liu, X., Lai, W., Liu, Y., Xiao, D., et al. (2019). DNA methylation modifier LSH inhibits p53 ubiquitination and transactivates p53 to promote lipid metabolism. Epigenetics Chromatin *12*, 59.

24. Sui, S., Zhang, J., Xu, S., Wang, Q., Wang, P., and Pang, D. (2019). Ferritinophagy is required for the induction of ferroptosis by the bromodomain protein BRD4 inhibitor (+)-JQ1 in cancer cells. Cell Death Dis. *10*, 331.

25. Galluzzi, L., Vitale, I., Aaronson, S.A., Abrams, J.M., Adam, D., Agostinis, P., Alnemri, E.S., Altucci, L., Amelio, I., Andrews, D.W., et al. (2018). Molecular mechanisms of cell death: recommendations of the Nomenclature Committee on Cell Death 2018. Cell Death Differ. *25*, 486–541.

26. Tang, D., Kang, R., Berghe, T.V., Vandenabeele, P., and Kroemer, G. (2019). The molecular machinery of regulated cell death. Cell Res. *29*, 347–364.

27. Zhou, B., Liu, J., Kang, R., Klionsky, D.J., Kroemer, G., and Tang, D. (2019). Ferroptosis is a type of autophagy-dependent cell death. Semin. Cancer Biol. Published online March 14, 2019. https://doi.org/10.1016/j.semcancer.2019.03.002.

28. Kain, H.S., Glennon, E.K.K., Vijayan, K., Arang, N., Douglass, A.N., Fortin, C.L., Zuck, M., Lewis, A.J., Whiteside, S.L., Dudgeon, D.R., et al. (2020). Liver stage malaria infection is controlled by host regulators of lipid peroxidation. Cell Death Differ. *27*, 44–54.

29. Huang, C., Yang, M., Deng, J., Li, P., Su, W., and Jiang, R. (2018). Upregulation and activation of p53 by erastin-induced reactive oxygen species contribute to cytotoxic and cytostatic effects in A549 lung cancer cells. Oncol. Rep. *40*, 2363–2370.

30. Roh, J.L., Kim, E.H., Jang, H.J., Park, J.Y., and Shin, D. (2016). Induction of ferroptotic cell death for overcoming cisplatin resistance of head and neck cancer. Cancer Lett. *381*, 96–103.

31. Fearnhead, H.O., Vandenabeele, P., and Vanden Berghe, T. (2017). How do we fit ferroptosis in the family of regulated cell death? Cell Death Differ. *24*, 1991–1998.

32. Kothari, A., Hittelman, W.N., and Chambers, T.C. (2016). Cell Cycle-Dependent Mechanisms Underlie Vincristine-Induced Death of Primary Acute Lymphoblastic Leukemia Cells. Cancer Res. *76*, 3553–3561.

33. Diouf, B., Crews, K.R., Lew, G., Pei, D., Cheng, C., Bao, J., Zheng, J.J., Yang, W., Fan, Y., Wheeler, H.E., et al. (2015). Association of an inherited genetic variant with vincristine-related peripheral neuropathy in children with acute lymphoblastic leukemia. JAMA *313*, 815–823.

34. Gao, W.Z., Guo, L.M., Xu, T.Q., Yin, Y.H., and Jia, F. (2018). Identification of a multidimensional transcriptome signature for survival prediction of postoperative glioblastoma multiforme patients. J. Transl. Med. *16*, 368.

35. Gatliff, J., East, D., Crosby, J., Abeti, R., Harvey, R., Craigen, W., Parker, P., and Campanella, M. (2014). TSPO interacts with VDAC1 and triggers a ROS-mediated inhibition of mitochondrial quality control. Autophagy *10*, 2279–2296.

36. Tarangelo, A., Magtanong, L., Bieging-Rolett, K.T., Li, Y., Ye, J., Attardi, L.D., and Dixon, S.J. (2018). p53 Suppresses Metabolic Stress-Induced Ferroptosis in Cancer Cells. Cell Rep. *22*, 569–575.

37. Cao, J.Y., Poddar, A., Magtanong, L., Lumb, J.H., Mileur, T.R., Reid, M.A., Dovey, C.M., Wang, J., Locasale, J.W., Stone, E., et al. (2019). A Genome-wide Haploid Genetic Screen Identifies Regulators of Glutathione Abundance and Ferroptosis Sensitivity. Cell Rep. *26*, 1544–1556.e8.

38. Wang, R., Shi, Y., Chen, L., Jiang, Y., Mao, C., Yan, B., Liu, S., Shan, B., Tao, Y., and Wang, X. (2015). The ratio of FoxA1 to FoxA2 in lung adenocarcinoma is regulated by LncRNA HOTAIR and chromatin remodeling factor LSH. Sci. Rep. *5*, 17826.

39. Termanis, A., Torrea, N., Culley, J., Kerr, A., Ramsahoye, B., and Stancheva, I. (2016). The SNF2 family ATPase LSH promotes cell-autonomous de novo DNA methylation in somatic cells. Nucleic Acids Res. *44*, 7592–7604.

40. Chu, C., Zhang, Q.C., da Rocha, S.T., Flynn, R.A., Bharadwaj, M., Calabrese, J.M., Magnuson, T., Heard, E., and Chang, H.Y. (2015). Systematic discovery of Xist RNA binding proteins. Cell *161*, 404–416.

41. Bester, A.C., Lee, J.D., Chavez, A., Lee, Y.R., Nachmani, D., Vora, S., Victor, J., Sauvageau, M., Monteleone, E., Rinn, J.L., et al. (2018). An Integrated Genome-wide CRISPRa Approach to Functionalize lncRNAs in Drug Resistance. Cell *173*, 649–664.e20.

42. Liu, B., Ma, X., Liu, Q., Xiao, Y., Pan, S., and Jia, L. (2018). Aberrant mannosylation profile and FTX/miR-342/ALG3-axis contribute to development of drug resistance in acute myeloid leukemia. Cell Death Dis. *9*, 688.

43. Sun, Q.L., Zhao, C.P., Wang, T.Y., Hao, X.B., Wang, X.Y., Zhang, X., and Li, Y.C. (2015). Expression profile analysis of long non-coding RNA associated with vincristine resistance in colon cancer cells by next-generation sequencing. Gene *572*, 79–86.

44. Wang, Y., Zhang, D., Wu, K., Zhao, Q., Nie, Y., and Fan, D. (2014). Long noncoding RNA MRUL promotes ABCB1 expression in multidrug-resistant gastric cancer cell sublines. Mol. Cell. Biol. *34*, 3182–3193.

45. Chang, L., Hu, Z., Zhou, Z., and Zhang, H. (2018). Linc00518 Contributes to Multidrug Resistance Through Regulating the MiR-199a/MRP1 Axis in Breast Cancer. Cell. Physiol. Biochem. *48*, 16–28.

46. Garzon, R., Volinia, S., Papaioannou, D., Nicolet, D., Kohlschmidt, J., Yan, P.S., Mrózek, K., Bucci, D., Carroll, A.J., Baer, M.R., et al. (2014). Expression and prognostic impact of lncRNAs in acute myeloid leukemia. Proc. Natl. Acad. Sci. USA *111*, 18679–18684.

47. Lyu, Y., Lou, J., Yang, Y., Feng, J., Hao, Y., Huang, S., Yin, L., Xu, J., Huang, D., Ma, B., et al. (2017). Dysfunction of the WT1-MEG3 signaling promotes AML leukemogenesis via p53-dependent and -independent pathways. Leukemia *31*, 2543–2551.

48. Lee, D.W., Zhang, K., Ning, Z.Q., Raabe, E.H., Tintner, S., Wieland, R., Wilkins, B.J., Kim, J.M., Blough, R.I., and Arceci, R.J. (2000). Proliferation-associated SNF2-like gene (PASG): a SNF2 family member altered in leukemia. Cancer Res. *60*, 3612–3622.

49. Han, Y., Ren, J., Lee, E., Xu, X., Yu, W., and Muegge, K. (2017). Lsh/HELLS regulates self-renewal/proliferation of neural stem/progenitor cells. Sci. Rep. *7*, 1136.

50. Deng, F., Sharma, I., Dai, Y., Yang, M., and Kanwar, Y.S. (2019). Myo-inositol oxygenase expression profile modulates pathogenic ferroptosis in the renal proximal tubule. J. Clin. Invest. *129*, 5033–5049.

51. Xia, X., Fan, X., Zhao, M., and Zhu, P. (2019). The Relationship between Ferroptosis and Tumors: A Novel Landscape for Therapeutic Approach. Curr. Gene Ther. *19*, 117–124.

52. Wang, G.X., Tu, H.C., Dong, Y., Skanderup, A.J., Wang, Y., Takeda, S., Ganesan, Y.T., Han, S., Liu, H., Hsieh, J.J., and Cheng, E.H. (2017). ΔNp63 Inhibits Oxidative Stress-Induced Cell Death, Including Ferroptosis, and Cooperates with the BCL-2 Family to Promote Clonogenic Survival. Cell Rep. *21*, 2926–2939.

53. Doll, S., Freitas, F.P., Shah, R., Aldrovandi, M., da Silva, M.C., Ingold, I., Goya Grocin, A., Xavier da Silva, T.N., Panzilius, E., Scheel, C.H., et al. (2019). FSP1 is a glutathione-independent ferroptosis suppressor. Nature *575*, 693–698.

54. Bersuker, K., Hendricks, J.M., Li, Z., Magtanong, L., Ford, B., Tang, P.H., Roberts, M.A., Tong, B., Maimone, T.J., Zoncu, R., et al. (2019). The CoQ oxidoreductase FSP1 acts parallel to GPX4 to inhibit ferroptosis. Nature *575*, 688–692.

55. Tang, Y., Wang, J., Lian, Y., Fan, C., Zhang, P., Wu, Y., Li, X., Xiong, F., Li, X., Li, G., et al. (2017). Linking long non-coding RNAs and SWI/SNF complexes to chromatin remodeling in cancer. Mol. Cancer *16*, 42.

56. Zhu, Y., Rowley, M.J., Böhmdorfer, G., and Wierzbicki, A.T. (2013). A SWI/SNF chromatin-remodeling complex acts in noncoding RNA-mediated transcriptional silencing. Mol. Cell *49*, 298–309.

57. Raj, A., van den Bogaard, P., Rifkin, S.A., van Oudenaarden, A., and Tyagi, S. (2008). Imaging individual mRNA molecules using multiple singly labeled probes. Nat. Methods *5*, 877–879.

58. Xiao, D., Shi, Y., Fu, C., Jia, J., Pan, Y., Jiang, Y., Chen, L., Liu, S., Zhou, W., Zhou, J., and Tao, Y. (2016). Decrease of TET2 expression and increase of 5-hmC levels in myeloid sarcomas. Leuk. Res. *42*, 75–79.

YMTHE, Volume *29*

Supplemental Information

A Nuclear Long Non-Coding RNA LINC00618 Accelerates Ferroptosis in a Manner Dependent upon Apoptosis

Zuli Wang, Xiaowen Chen, Na Liu, Ying Shi, Yating Liu, Lianlian Ouyang, Samantha Tam, Desheng Xiao, Shuang Liu, Feiqiu Wen, and Yongguang Tao

Supplementary Figures

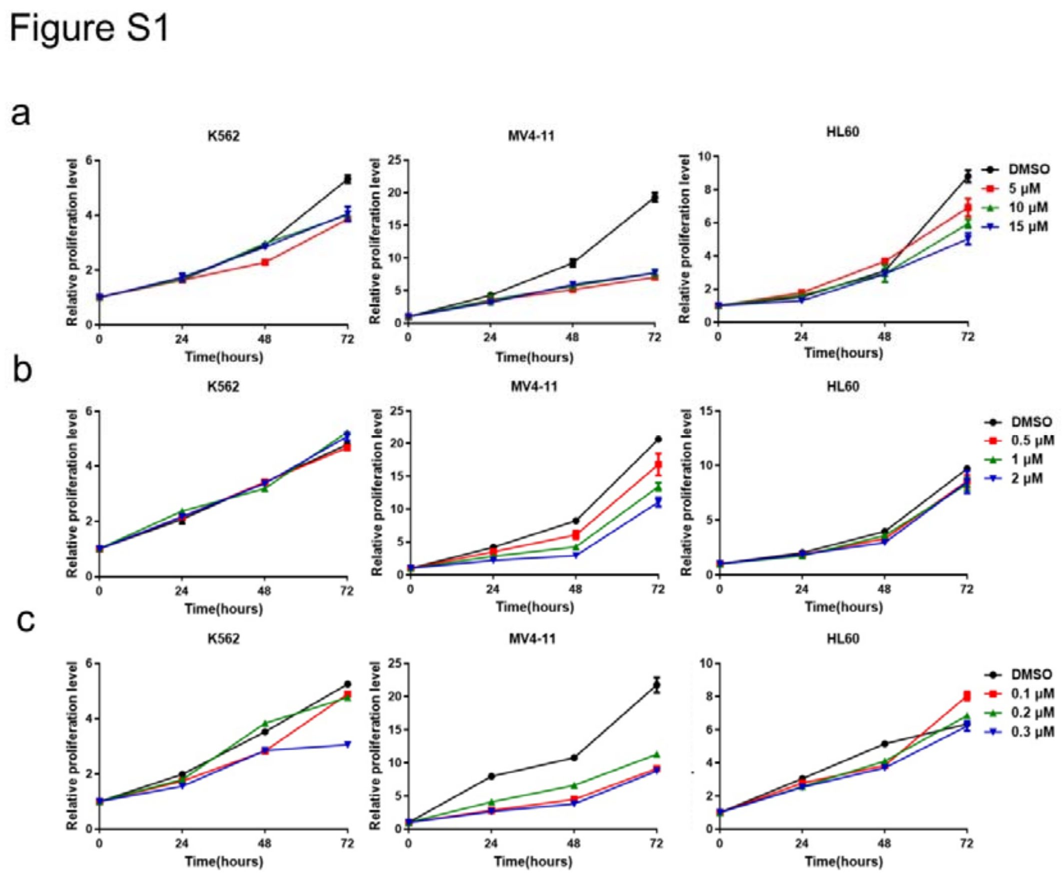

Figure. S1. 6-mercatopurine monohydrate, Imatinib Mesylatem and AZD9496 affecting cell growth.

a The relative proliferation levels of K562, HL60 and MV4-11 cells checked by MTS assays with the indicated doses of 6-Mercatopurine Monohydrate treatment. DMSO served as control. **b** The relative proliferation levels of K562, HL60 and MV4-11 cells checked by MTS assays with the indicated doses of Imatinib Mesylate treatment. DMSO served as control. **c** The relative proliferation levels of K562, HL60 and MV4-11 cells checked by MTS assays with the indicated doses of AZD9496 treatment. DMSO served as control.

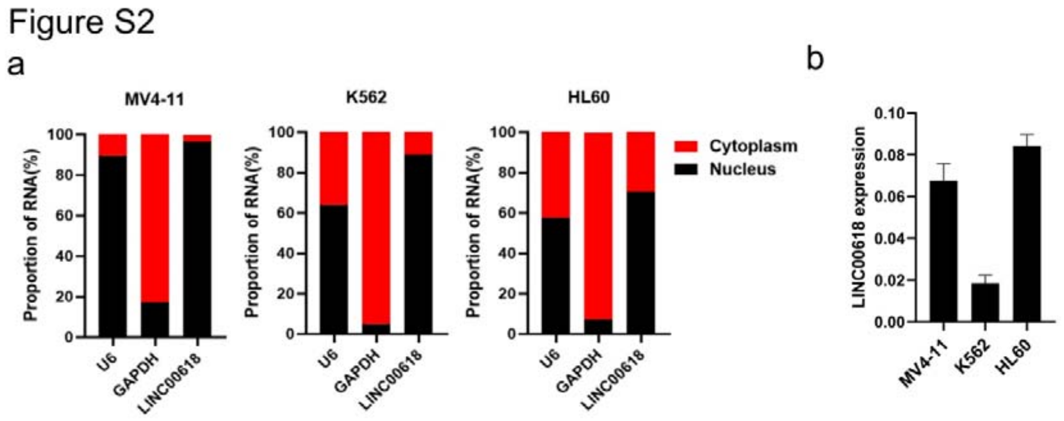

Figure. S2. The expression and localization of LINC00618 in MV4-11, K562 and HL60 cells.

a The RNA expression level of LINC00618 in MV4-11, K562 and HL60 cells. **b** The LINC00618 expression level in the nuclear and cytosolic fractions derived from K562 cells and HL60 cells

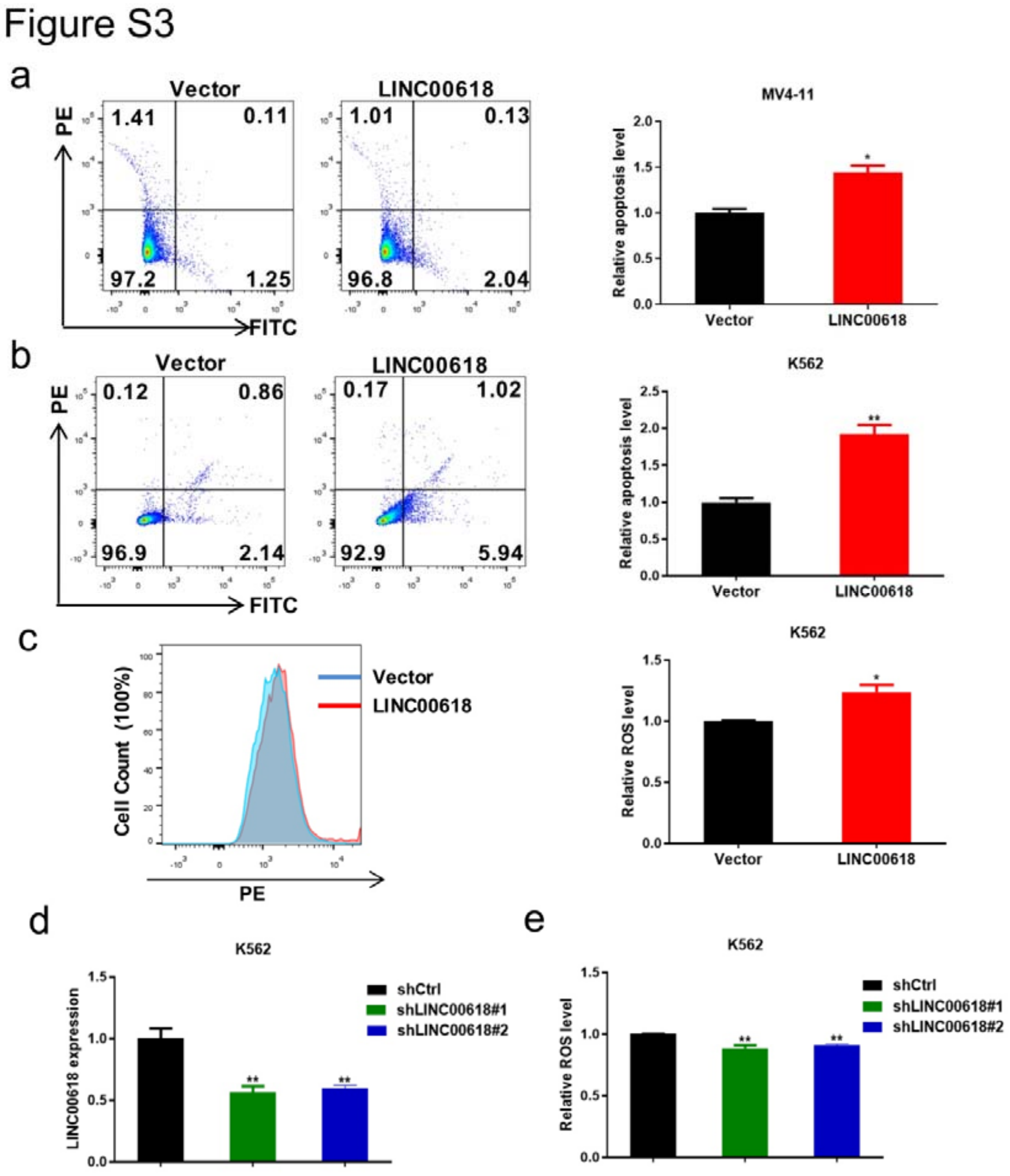

Figure. S3. The role of LINC00618 in promoting cell apoptosis and ferroptosis.

a Flow cytometry and statistical analysis showed that LINC00618 promoted apoptosis in MV4-11 cells stably overexpressing LINC00618. **b** Flow cytometry and statistical analysis showed that LINC00618 promoted apoptosis in K562 cells stably overexpressing LINC00618. **c** Flow cytometry and statistical analysis of ROS level in K562 cells stably overexpressing LINC00618. **d** qRT-PCR analyses of stably knockdown vector and LINC00618 in K562 cells. **e** Flow cytometry and statistical

analysis of ROS level in LINC00618-knockdown K562 cells.

Data are shown as the mean ± SEM; $n \geq 3$ independent experiments, two-tailed Student's t-test: ns nonsignificant ($p > 0.05$), *$P < 0.05$, **$P < 0.01$, ***$P < 0.001$.

Figure S4

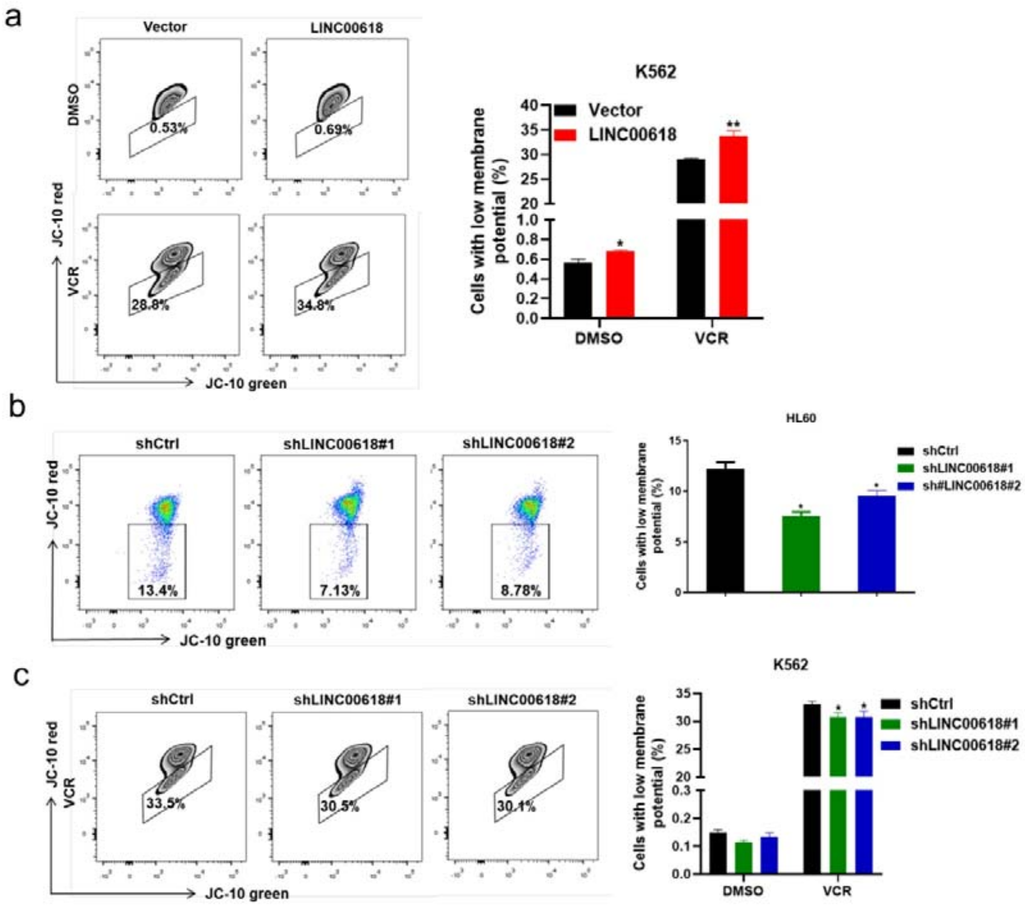

Figure. S4. The role of LINC00618 in mitochondrial membrane potential.

a Flow cytometry and statistical analysis of mitochondrial membrane potential using JC-10 in LINC00618-overexpressed K562 cells treated with VCR. DMSO served as control. **b** Flow cytometry and statistical analysis of mitochondrial membrane potential using JC-10 in LINC00618-konckdown HL60 cells. **c** Flow cytometry and statistical analysis of mitochondrial membrane potential using JC-10 in LINC00618-knockdown K562 cells treated with VCR. DMSO served as control.

Figure S5

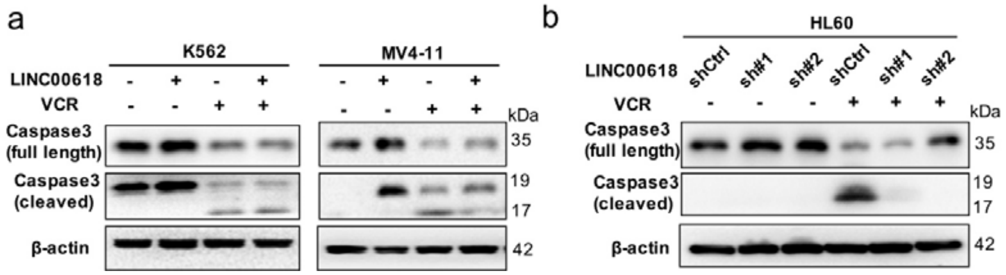

Figure. S5. The effect of LINC00618 on apoptosis related proteins.

a Expression level of apoptosis related proteins were measured by western blot in K562 and MV4-11 cells stably overexpressing LINC00618. **b** Expression level of apoptosis related proteins were measured by western blot in HL60 cells stably interfering LINC00618.

Figure S6

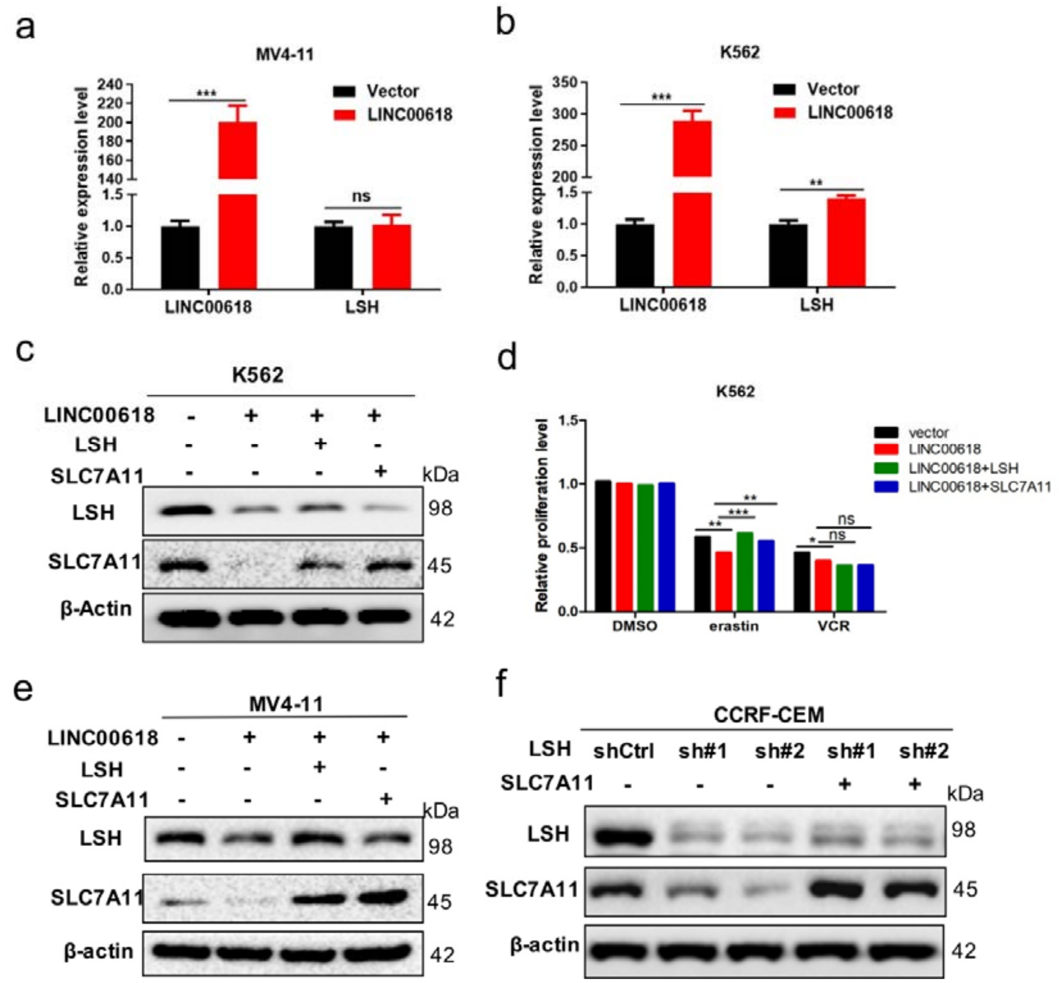

Figure. S6. SLC7A11 is regulated by LINC00618 through LSH.

a qRT-PCR analyses of LSH in MV4-11 cells stably overexpressing LINC00618. **b** qRT-PCR analyses of LSH in K562 cells stably overexpressing LINC00618. **c** Western blot analyses of LSH and SLC7A11 in K562 cells stably overexpressing LINC00618 with LSH or SLC7A11 overexpression. **d** MTS assay analyses of relative proliferation levels of K562 cell lines with erastin or VCR treatment from **c.** DMSO served as control. **d** **W**estern blot analyses of LSH and SLC7A11 in MV4-11 cells stably overexpressing LINC00618 with LSH or SLC7A11 overexpression. **e** Western blot analyses of LSH and SLC7A11 in CCRF-CEM cells after the knockdown of LSH

with SLC7A11 overexpression.

Supplementary Tables

Table S1. Primer sequences used for RT-PCR

Name	Full Name	Gene ID	sequence	Product Size(bp)
LINC00618	long intergenic non-protein coding RNA 618	145249	F: AAGGGGTGGGGACACGTTG<br>R: TCTCTCTCCGTCAGCCTCAG	140
LSH	Lymphoid-specific helicase	3070	F: GATTTTGGATCGAATGCTGCCAG<br>R: ATGGACCCATCAAGCCTGCTGA	141
SLC7A11	solute carrier family 7 member 11	23657	F: TCCTGCTTTGGCTCCATGAACG<br>R: AGAGGAGTGTGCTTGCGGACAT	122
GPX4	glutathione peroxidase 4	2879	F: ACAAGAACGGCTGCGTGGTGAA<br>R: GCCACACACTTGTGGAGCTAGA	126
ACSL4	acyl-CoA synthetase long chain family member 4	2182	F: GCTATCTCCTCAGACACACCGA<br>R: AGGTGCTCCAACTCTGCCAGTA	139
P53	Tumor protein p53	7157	F: CCTCAGCATCTTATCCGAGTGG<br>R: TGGATGGTGGTACAGTCAGAGC	128
CS	citrate synthase	1431	F: CACAGGGTATCAGCCGAACCAA<br>R: CCAATACCGCTGCCTTCTCTGT	127
ATP5G3	ATP synthase membrane subunit c locus 3	518	F: GCAACAGTAGGAGTGGCTGGTT<br>R: GCTTCAGACAAGGCAAATCCCAG	137
CBS	cystathionine-beta-synthase	875	F: CATTGCCAGGAAGCTGAAGGAG<br>R: TTCCACCTCGTAGGTTGTCTGC	124
HMGCR	3-hydroxy-3-methylglutaryl -CoA reductase	3156	F: GACGTGAACCTATGCTGGTCAG<br>R: GGTATCTGTTTCAGCCACTAAGG	119
LPCAT3	lysophosphatidylcholine acyltransferase 3	10162	F: CAGGATACCTGGTCTGCTTCCA<br>R: TGAAGAGCCAGTGGATGGTCTG	162
TFRC	transferrin receptor	7037	F: ATCGGTTGGTGCCACTGAATGG<br>R: ACAACAGTGGGCTGGCAGAAAC	131

Table S2. Primer sequences used for ChIP

Name	Sequence	Product size(bp)
SLC7A11#1	F: AGTTGGTGTGACAGGCAGGCGCTTA	122
	R: TCTCAATTCTCCACCTCCTCGTTCC	
SLC7A11#2	F: GGAACGAGGAGGTGGAGAATTGAGA	123
	R: CTCAGCGCTATAGTGTTCACAGGTG	

Table S3. Probe sequences used for ChIRP

Name	Sequence	modification
LINC00618#1	CACCATGGATGCAACGTGTC	3`Biotin-TEG
LINC00618#2	TTCATCTCTGGAAACTGCCC	3`Biotin-TEG
LINC00618#3	TGGCTTCGATCCAGAGTGTC	3`Biotin-TEG
